# Double functionalized haemocompatible silver nanoparticles control cell inflammatory homeostasis

**DOI:** 10.1371/journal.pone.0276296

**Published:** 2022-10-21

**Authors:** Mamta Kumawat, Harishkumar Madhyastha, Mandeep Singh, Neerish Revaprasadu, Sangly P. Srinivas, Hemant Kumar Daima

**Affiliations:** 1 Amity Center for Nanobiotechnology and Nanomedicine (ACNN), Amity Institute of Biotechnology, Amity University Rajasthan, Jaipur, Rajasthan, India; 2 Department of Cardiovascular Physiology, Faculty of Medicine, University of Miyazaki, Miyazaki, Japan; 3 School of Science, RMIT University, Melbourne, Victoria, Australia; 4 Department of Chemistry, University of Zululand, Richards Bay, South Africa; 5 School of Optometry, Indiana University, Bloomington, IN, United States of America; Brandeis University, UNITED STATES

## Abstract

Infection, trauma, and autoimmunity trigger tissue inflammation, often leading to pain and loss of function. Therefore, approaches to control inflammation based on nanotechnology principles are being developed in addition to available methods. The metal-based nanoparticles are particularly attractive due to the ease of synthesis, control over physicochemical properties, and facile surface modification with different types of molecules. Here, we report curcumin conjugated silver (Cur-Ag) nanoparticles synthesis, followed by their surface functionalization with isoniazid, tyrosine, and quercetin, leading to Cur-Ag^INH^, Cur-Ag^Tyr^, and Cur-Ag^Qrc^ nanoparticles, respectively. These nanoparticles possess radical scavenging capacity, haemocompatibility, and minimal cytotoxicity to macrophages. Furthermore, the nanoparticles inhibited the secretion of pro-inflammatory cytokines such as interleukin-6, tumor necrosis factor-α, and interleukin-1β from macrophages stimulated by lipopolysaccharide (LPS). The findings reveal that the careful design of surface corona of nanoparticles could be critical to increasing their efficacy in biomedical applications.

## 1. Introduction

Reactive oxygen species (ROS) are highly active, short-lived, small molecules, which are essential in signaling functions, and regulation of physiological activities (e.g., cell growth, differentiation, senescence, or apoptosis) in living cells. However, excessive production of ROS results in oxidative damage in cells/tissues, leading to the progression of inflammatory diseases [1, 2]. Inflammation, per se, is a defensive mechanism triggered by infection and injury against foreign pathogens. While acute inflammation mainly involves prostaglandins, chronic inflammation entails numerous inflammatory cytokines, including IL-1 and TNF-α [1, 3, 4]. In addition, specific ROS species behave pro-inflammatory by activating c-Jun N-terminal kinase (JNK), protein kinase C, growth factor tyrosine kinase receptor, and extracellular signal-regulated kinase signaling pathways [3].

Clinically, inflammation is managed through non-steroidal anti-inflammatory drugs (NSAIDs) and glucocorticoids for acute and chronic inflammation. However, high dosages and prolonged use of these drugs have numerous side effects. Thus, it is imperative to find alternatives to the chronic and sole use of NSAIDs and glucocorticoids. In this context, plant-derived phytochemicals are helpful because of their lower toxicity. However, adverse events may arise due to incorrect species of medicinal plants or dosing and interaction with other drugs. Therefore, alternative approaches for new anti-inflammatory agents with sustained release and better efficacy are sought. In this direction, nanotechnology offers promise in drug development with enhanced efficacy and controlled drug release.

In the past few decades, tremendous interest has been reported in biomedical research of metallic nanoparticles such as silver (Ag) and gold (Au). These can reduce oxidative stress through their efficient radical scavenging activities. Ag-based nanoparticles also confer a broad range of antimicrobial activity and are used in various wound dressings to control bacterial infections. They are also well known for anti-fungal, anti-inflammatory, and antioxidant activities [5, 6]. Thus, Ag nanoparticles have contributed significantly to the field of biomedicine. However, the nanoparticle synthesis route is vital to extend their full potential, and hence it is crucial to develop monodispersed particles and fine-tune their physicochemical properties to reduce toxicity [7–9].

The biosynthetic methods involving phytochemicals enable novel opportunities in reducing Ag ions to form Ag nanoparticles. Alkaloids, flavonoids, and anthraquinones are natural active components of curcumin (Curcuma longa), a plant-based natural lipophilic compound [10]. Curcumin shows anti-inflammatory, antioxidant, anti-cancer, and anti-bacterial activities [11]. In addition, it targets various transcription factors and inhibits the production of pro-inflammatory cytokines, including interleukin 6/8 and TNF-α [12]. It is also an inducer of cell death via extrinsic and intrinsic apoptosis pathways and regulates gene expression [13]. However, curcumin’s efficacy is limited because of its poor water solubility, low absorption, and rapid degradation. These problems can be surmounted by loading or conjugating curcumin on the surface of nanoparticles [14, 15]. In addition, the release of hydrogen from curcumin shows a reduction of metal ions and thus acts precursor for nanoparticle formation [16], whereas nanoparticle’s utility in biomedicine depends upon their surface modification with drugs, biomolecules, ligands, or amino acids [17–22].

In this study, we report curcumin-based Ag nanoparticles (Cur-Ag) synthesis, followed by their surface alteration with isonicotinic acid hydrazide (INH, isoniazid), tyrosine (Tyr, 4-hydroxyphenylalanine), and quercetin (Qrc, plant flavonol) to formulate Cur-Ag^INH^, Cur-Ag^Tyr^, and Cur-Ag^Qrc^ nanoparticles, respectively. These molecules were selected based on their potential biomedical capabilities. Thus, isoniazid is an antibiotic, tyrosine is an amino acid of biological origin, and quercetin is an antioxidant. Isoniazid is the first-line antibiotic used to treat *Mycobacterium* infection by inhibiting mycolic acid, a structural component of the *Mycobacterium* cell envelope. Isoniazid surface-modified particles can enhance the bioavailability of the drug in treating tuberculosis [23, 24]. Conjugation of isoniazid with other molecules, including curcumin, can lower hepatotoxicity, improve stability, and enhance bioavailability [25].

Similarly, amino acid-based surface corona can offer a natural identity to nanoparticles. Thus, tyrosine is a proteinogenic amino acid [26], and its presence on the nanoparticle’s surface can provide an additional biological identity to the particle. Moreover, due to the zwitterionic nature of amino acids, the surface charge of nanoparticles can be easily tailored by varying the solution pH [27, 28]. Likewise, quercetin is vital for antioxidant, anti-cancer, anti-inflammatory, and antiviral activities [29]. However, its poor water solubility, chemical instability, and low bioavailability limit the biomedical application of quercetin. To overcome these issues, modification, and further enhancement of efficacy in the nano-formulation-based system have been accomplished, and the nanoscale size material can facilitate their interaction with various biomolecules [30]. Hence, surface functionalization of Cur-Ag nanoparticles with isoniazid, tyrosine, and quercetin can provide unique opportunities to control the cell inflammatory haemostasis balance due to the involvement of diverse surface chemistries.

## 2. Experimental section

### 2.1. Materials and regents

Curcumin, dimethyl sulphoxide (DMSO), ethylenediaminetetraacetic acid (EDTA), hydrogen peroxide (H_2_O_2_) 30% (w/v), isoniazid, phosphate-buffered saline (PBS), lipopolysaccharides (LPS), potassium hydroxide (KOH), potassium persulfate (K_2_S_2_O_8_), quercetin, silver nitrate (AgNO_3_), tyrosine, 2,2′-azino-bis (3-ethylbenzothiazoline-6-sulphonic acid) (ABTS), and 2`-7`-Dichlorofluorescin diacetate (DCFH-DA) were procured from Sigma Aldrich, St. Louis Missouri, United States. Dialysis membrane (LA 398-5MT) was acquired from Hi-media laboratory, Mumbai; sodium dodecyl sulfate (SDS) from Merck, and sodium chloride from Fisher Scientific, Mumbai, respectively. Dulbecco`s modified Eagle medium (DMEM) has low glucose and 10% heat-inactivated fetal bovine serum from Sigma Aldrich, USA, with an antibiotic concoction (5 mg/mL penicillin, 5 mg/mL streptomycin, and 10 mg/mL neomycin from Gibco Inc, Tokyo, Japan). Radioimmunoprecipitation (RIPA) cell lysis buffer from Thermo Scientific, Carlsbad, USA, sodium dodecyl sulfate-polyacrylamide gel electrophoresis (SDS-PAGE) gels, polyvinylidene fluoride (PVDF) membrane from Immuno Blot, Bio-Rad, California, USA, blotted membrane from EzBlock Chemi, Atto, Corp, Japan, primary antibodies; anti-rabbit IL-6, anti-rabbit TNF-α, anti-rabbit- IL1β, anti-rabbit β-actin from cell signaling Technology Inc, Danvers, MA, USA, tris buffered saline (T-TBS) membranes with horseradish peroxidase (HRP) conjugated secondary antibody, and Enhanced chemiluminescence (ECL) reagents from GE health care, Tokyo, Japan, were procured and used as instructed.

The Raw 264.7 macrophage cells from RIKEN cell bank of RIEKN cell engineering division, Tsukuba, Ibaraki, Japan; RNAiso reagents from Takara Biolabs, Tokyo, Japan. OxiSelect^TM^ intracellular ROS assay Kit from Cell BioLabs, Inc, San Diego, CA, USA. Semi-quantitative PCR (q-PCR) by DreamTaq Green PCR master mix from Thermo Fisher Scientific, Waltham, MA, USA, and bicinchoninic acid (BCA) protein assay kits from Thermo Scientific, Carlsbad, USA. All the chemicals and reagents were used as received according to manufacturer instructions. Ultrapure milli-Q water was used throughout the study with a resistivity of 18.2 MΩ cm at 25°C. All the glassware used to prepare nanoparticles were thoroughly washed with aqua-regia (a mixture of concentrated HCI and HNO_3_).

### 2.2. Curcumin-mediated synthesis of Ag nanoparticles and their surface functionalization

In a typical experiment, Cur-Ag nanoparticles were synthesized using curcumin as a reducing and stabilizing agent. First, in a 50 mL aqueous solution, 1 mM KOH and 0.25 mM curcumin were allowed to heat, followed by dropwise addition of 1 mM of AgNO_3_ under constant stirring. After adding AgNO_3_ (within 5–10 mins), a yellowish-brown color developed, indicating the formation of Cur-Ag nanoparticles. Next, to increase the metal content (2x) in the solution, Cur-Ag nanoparticles were subjected to slow heating with constant stirring. Additionally, the concentrated Cur-Ag nanoparticles solution was dialyzed using a dialysis membrane to remove the unreacted molecules and ions against Milli-Q water for 3 h at room temperature. Later, the concentrated, and dialyzed Cur-Ag nanoparticles, further surface functionalized with 0.25 mM of isoniazid, tyrosine, or quercetin to produce Cur-Ag^INH^, Cur-Ag^Tyr,^ and Cur-Ag^Qrc^, respectively. After successful surface modification, these solutions were again subjected to dialysis to remove any unbound molecules from the surfaces against Milli-Q water.

### 2.3. Physicochemical characterization of nanoparticles

The Cur-Ag, Cur-Ag^INH^, Cur-Ag^Tyr^_,_ and Cur-Ag^Qrc^ nanoparticles were characterized by UV-Visible spectroscopy (Biomate 3S, ThermoFisher, USA), Fourier-transform infrared spectroscopy (Thermo Scientific NICOLET iS5, USA), transmission electron microscopy (JEOL1010), atomic absorption spectroscopy (Shimadzu AA 7000, Tokyo, Japan), and zeta-sizer (version V2.3, Malvern, UK). Interactions of nanoparticles with cells characterized by confocal laser scanning microscope TCS Sp8 (Lieca Optical Inc, Wetzlar, Germany), agarose gel electrophoresis (BioRad Inc, Tokyo, Japan), bands detection using BioRad digital imaging system (BioRad Inc, Tokyo, Japan), and band intensity analyzed by ImageQuant TL software (GE, Healthcare Life Science, IL, USA).

### 2.4. Assessment of radical scavenging capacity (RSC) of nanoparticles

ABTS assay was performed to determine the RSC of nanoparticles. Three different concentrations of Cur-Ag, Cur-Ag^INH^, Cur-Ag^Tyr^, and Cur-Ag^Qrc^ nanoparticles (0.0412 mmol/L, 0.0618 mmol/L, and 0.0824 mmol/L) were allowed to react with ABTS^·+^. A decrease in the absorbance was measured at 734 nm to calculate the percentage RSC. ABTS^·+^ radicals were formed by reacting 7.4 mM of ABTS and 2.45 mM of potassium persulphate in the ratio of (2:1) by keeping them in the dark for 12–16 h at room temperature. The color change is an indication of radical formation. These pre-formed ABTS^·+^ radicals were further diluted to achieve an absorbance of 0.67±0.02 with absolute ethanol at 734 nm. The formed ABTS^·+^ radical cation is inhibited by the antioxidant nature of nanoparticles [31, 32].

### 2.5. Haemocompatibility of nanoparticles

The effects of Cur-Ag, Cur-Ag^INH^, Cur-Ag^Tyr,^ and Cur-Ag^Qrc^ nanoparticles are assessed on red blood cells (RBCs) at 5 different concentrations of nanoparticles. The assay was performed using 5 mL of blood sample in a pre-EDTA stabilized tube and centrifuge at 10,000 rpm at 4°C for 10 min to separate blood components by settling RBC at the bottom. Then, the supernatant was discarded, and the pellet with RBCs was resuspended in 0.9% sodium chloride (isotonic to RBC) to make a suspension. Next, the nanoparticles were treated with five different concentrations (0.00412 mmol/L, 0.0206 mmol/L, 0.0412 mmol/L, 0.0618 mmol/L, and 0.0824 mmol/L) of nanoparticles with 2.1 mL of diluted RBC suspension and incubated for 3 h on a shaker at room temperature. After incubation, centrifugation was carried out at 10,000 rpm/3 min, and the haem release in the supernatant was measured at 540 nm to calculate the percentage of haemolysis. The same treatment was also given to positive and negative controls by taking 1% SDS and 0.9% sodium chloride with the respective volume of RBC suspension.

### 2.6. MTT assay

Cell proliferation of mouse Raw 264.7 macrophages was evaluated using MTT assay. The assay was performed by having cells from early (3–4) passage and cultured in Dulbecco`s modified Eagle low glucose medium with 10% heat-inactivated fetal bovine serum and antibiotic combination including (5 mg/mL penicillin, 5 mg/mL streptomycin, and 10 mg/mL neomycin) at 37°C with 5% CO_2_. Semi confluent cells (1x10^4^ cells/mL) were seeded and treated with different concentration 0.00 mmol/L, 0.0011 mmol/L, 0.0027 mmol/L, 0.0054 mmol/L, and 0.0082 mmol/L of nanoparticles and further incubated for the standard time of 16 h. The intracellular purple formazan was quantified with a UV-Vis spectrophotometer at an absorbance of 570 nm.

### 2.7. Intracellular ROS detection by fluorescence microscopy

The generation of intracellular ROS was measured by fluorescence microscopic method using the intracellular ROS assay Kit. 1x10^3^ cells/mL of mouse RAW 264.7 macrophages were seeded onto the culture suitable coverslips and treated with different nanoparticles for 16 h under a typical cell culture procedure. Following the treatment, 10 μM of 2`-7`-Dichlorofluorescin diacetate (DCFH-DA) was added at 37°C for 1 h. After treatment with DCFH-DA, cells were fixed with 4% paraformaldehyde and mounted on the cover glass. The degree of green-fluorescent intensity was observed at 20x under a confocal laser scanning microscope.

### 2.8. Cytokine expression assays

Semi-confluent Raw 264.7 macrophages were treated with 1 μg/mL of LPS with or without different test nanoparticles (Cur-Ag, and Cur-Ag^Tyr^, 0.0054 mmol/L at 24 h; Cur-Ag^INH^, and Cur-Ag^Qrc^, 0.0054 mmol/L at 12 h, and 16 h). After the treatment, total RNA was isolated using RNAiso reagents, and the first strand of cDNA was prepared. The transcript levels of the gene of interest were measured by semi-quantitative PCR (q-PCR) using the DreamTaq Green PCR master mix. Further, PCR products were electrophoresed on 1% agarose gel, and resulting bands were detected using the Bio-Rad digital imaging system. Band intensity was analyzed by ImageQuant TL software. β-actin was used as a housekeeping gene to normalize the individual inflammatory gene.

Following primers were used in the study of the inflammatory genes:

IL6: forward: 3`- CCGGAGCCGGAGAGGAGACTTCACAG -5`
TNF: forward: 3`-TACTGAACTTCGGGGTGATTGGTCC-5`
IL1β: forward: 3`- CACAGCAGCACATCAACAAG -5`

The mRNA expression profiles were further corroborated by Western blotting. Briefly, macrophages treated with LPS, and nanoparticles were lysed with cold RIPA cell lysis buffer, and a BCA protein assay kit was used to determine protein concentrations, followed by resolving 10 μg protein in 17% SDS-PAGE gels. Later, the separated protein was transferred to the PVDF membrane and blocked (blotted membrane) for 1 h after incubation with primary antibodies (anti-rabbit IL-6, anti-rabbit TNF-α, anti-rabbit IL-1β, anti-rabbit β- actin) at room temperature. In the end, these were probed with HRP conjugated secondary antibody (after washing with T-TBS membranes), and immunoreactivity of protein expression was detected.

### 2.9. Statistical analysis

One-way and two-way ANOVA (analysis of variance) with Tukey’s post-test analysis were used for statistical comparisons using the GraphPad Prism™ software (San Diego, CA; Version 5.0). Data are expressed as mean ± SE or mean ± SD. Unless specified otherwise, all experiments were performed in triplicates.

## 3. Results and discussion

**Fig 1:**
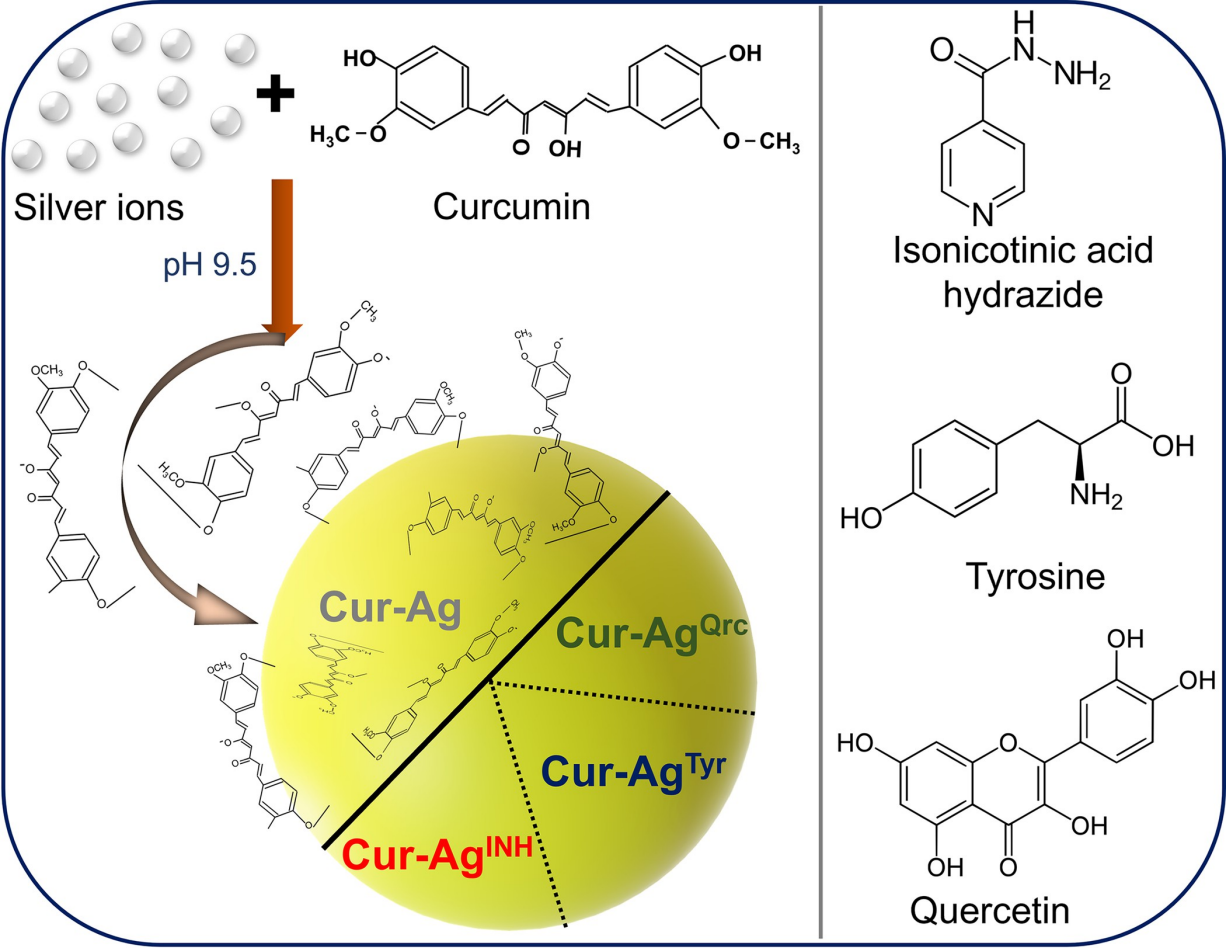
Formation of Cur-Ag nanoparticles at alkaline pH by reducing silver ions through curcumin. The particles were next treated with isonicotinic acid hydrazide (isoniazid), tyrosine, and quercetin to obtain corresponding functionalized nanoparticles (Cur-Ag^INH^, Cur-Ag^Tyr^, and Cur-Ag^Qrc^, respectively). The chemical structures of curcumin, isonicotinic acid hydrazide, tyrosine, and quercetin are also shown.

As illustrated in **Fig 1**, the present work focuses on preparing Ag nanoparticles with an aqueous solution of curcumin (reducing and stabilizing agent) and AgNO_3_ under an alkaline environment. After preparing Cur-Ag nanoparticles, they were functionalized using isoniazid, tyrosine, and quercetin to generate Cur-Ag^INH^, Cur-Ag^Tyr,^ or Cur-Ag^Qrc^ nanoparticles, respectively.

**Fig 2:**
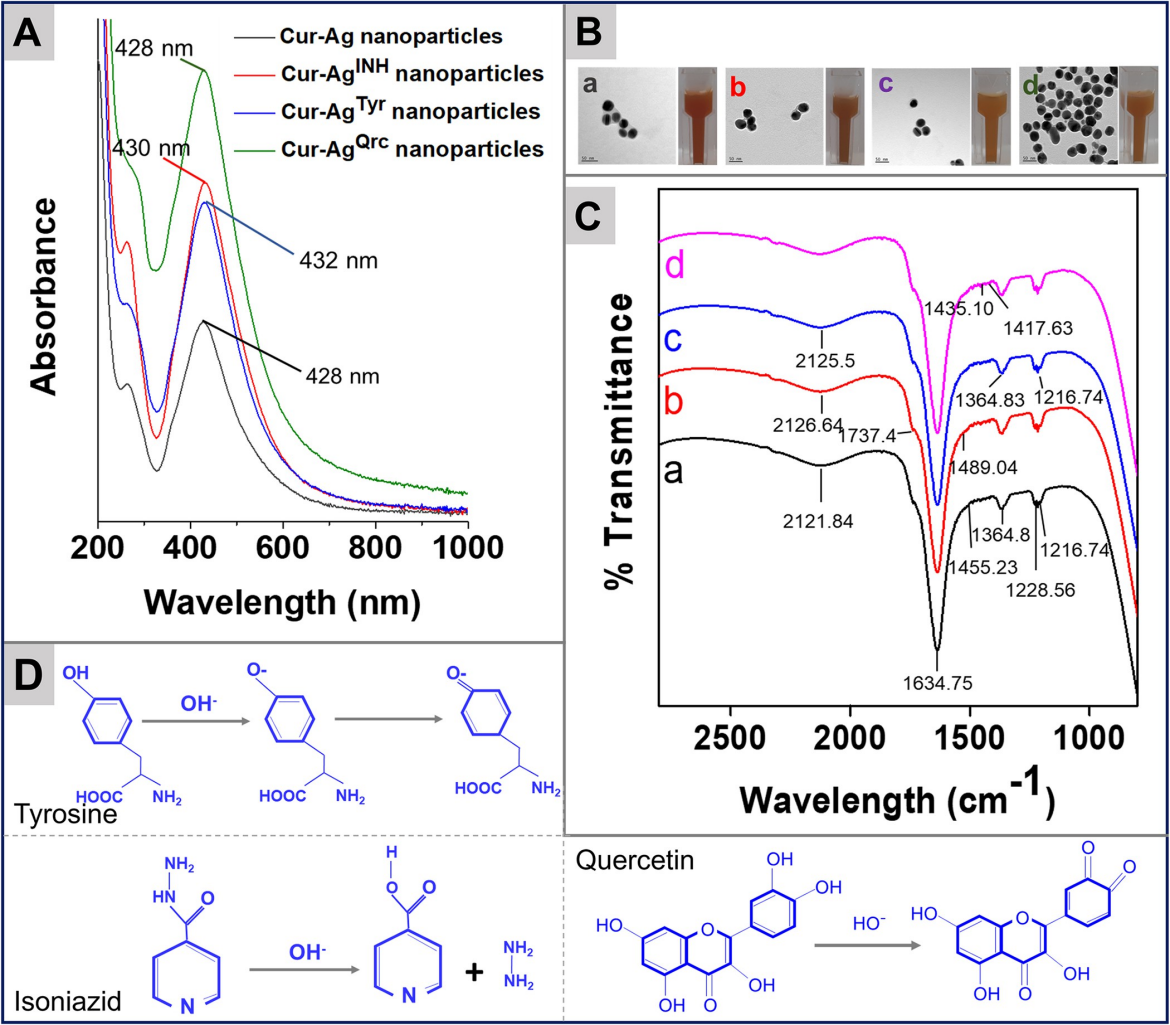
Physicochemical characterization of nanoparticles by UV-visible spectroscopy, TEM micrographs, nanoparticles solution images, and FTIR. Typical SPR absorbance bands of Ag nanoparticles (Panel A). Digital photographs of the nanoparticle’s solution and TEM micrographs of Cur-Ag (a), Cur-Ag^INH^ (b), Cur-Ag^Tyr^ (c), and Cur-Ag^Qrc^ (d) nanoparticles, respectively. The scale bar in TEM micrographs is 50 nm (Panel B). Critical vibrational frequencies of curcumin, isonicotinic acid hydrazide, tyrosine, and quercetin on the surface of Ag nanoparticles; a, b, c, and d correspond to Cur-Ag, Cur-Ag^INH^, Cur-Ag^Tyr^, and Cur-Ag^Qrc^ nanoparticles, respectively (Panel C). The chemical reactions of tyrosine, isoniazid, and quercetin molecules at alkaline pH at the surface of Cur-Ag nanoparticles (Panel D).

Curcumin (Cur) has three important functional groups, an aromatic methoxy phenolic group, α, β-unsaturated β-diketo linker, and keto-enol tautomerism. The keto and the phenolic groups are involved in H-bonding [33]. In an alkaline pH environment, the phenolate and enolate anions of curcumin help reduce Ag^+^ ions, thus producing Cur-Ag nanoparticles. Furthermore, at the alkaline pH, the nanoparticles formed show a high dispersity facilitated by a higher number of Ag(I) binding [34]. Next, surface functionalization was performed with isoniazid, tyrosine, and quercetin to obtain Cur-Ag^INH^, Cur-Ag^Tyr,^ or Cur-Ag^Qrc^ nanoparticles. The molecules for functionalization were selected based on their unique properties. Oxidized isoniazid, under alkaline pH conditions, binds on the surface of Cur-Ag nanoparticles as carboxylic acid binds to Ag [23, 24]. In tyrosine, the phenolic group gets converted into the semiquinone group and acts preferred site of binding on Cur-Ag nanoparticles [35, 36]. In addition, tyrosine’s aromatic moiety, C = O, and -NH_2_ functional groups are involved in binding. This binding interaction is mediated through the C = O of curcumin and the NH_2_ group of tyrosine [37]. Moreover, the aqueous solution has alkaline pH well above the isoelectric point of tyrosine (PI~5.66), indicating the overall negative surface charge, which was confirmed by zeta potential values [28]. In quercetin, the presence of phenolic group and H- donating ability promotes reduction. In the reduced form, the OH groups convert into the carbonyl group (C = O). These C = O groups in the oxidized form of polyphenol electrostatically stabilize the metal nanoparticles [38] and can attach to the surface of Cur-Ag nanoparticles to form Cur-Ag^Qrc^ nanoparticles. The chemical reactions for binding of tyrosine, isoniazid, and quercetin on the surface of Cur-Ag nanoparticles are shown in **Fig 2D**.

**Fig 2A and 2B** demonstrate the absorption spectra, optical images of nanoparticle solutions, and TEM micrographs of synthesized Cur-Ag and subsequent surface-functionalized Cur-Ag^INH^, Cur-Ag^Tyr,^ and Cur-Ag^Qrc^ nanoparticles. A good color change is an indication of AgNPs formation. The color changed from colorless to pale yellow at the start of curcumin, which intensified as the reaction proceeded and subsequently changed to yellow-brown with time, suggesting the development of Cur-Ag nanoparticles. This color change indicates the excitation of metal nanoparticle surface plasmon vibration [39]. In addition, variation in color intensity was observed from Cur-Ag nanoparticles to Cur-Ag^INH^, Cur-Ag^Tyr^, and Cur-Ag^Qrc^ nanoparticles indicating successful functionalization (**Fig 2B**).

Further, UV-visible spectroscopy was performed to confirm the synthesis and stability of nanoparticles in aqueous solutions. Cur-Ag nanoparticles gave surface plasmon resonance (SPR) at ~428 nm due to the π→π* transition, and an additional band at ~262 nm was also observed due to the n→π* transition from carbonyl and hydroxyl group [40, 41]. After surface modification of Cur-Ag nanoparticles with isoniazid, the SPR band red-shifted to ~432 nm. Similarly, the functionalization of Cur-Ag with tyrosine shifted the SPR to ~430 nm. However, when Cur-Ag nanoparticles are functionalized with quercetin, the values remain unchanged at ~428 nm, which might be due to the strong binding interactions of curcumin and quercetin molecules (**Fig 2A**). Similarly, as shown in **S1 Fig in S1 File**, the pyridine group of pure isoniazid exhibits an absorption band at ~262 nm due to π→π* transition [24], and the pristine tyrosine indicates an absorption band at ~224 nm owing to π→π* transitions of the peptide bonds [42], and quercetin at ~264 nm due to π→π* electronic transition of aromatic chromophore [43]. Finally, the TEM analysis was carried out to determine the morphology of nanoparticles, as shown in **Fig 2B(a-d)**. All the Cur-Ag, Cur-Ag^INH^, Cur-Ag^Tyr^, and Cur-Ag^Qrc^ nanoparticles are spherical to quasi-spherical in shapes with higher monodispersity. Moreover, there were no visible signs of aggregation in any of the nanoparticles solutions, indicating the stability of Cur-Ag, Cur-Ag^INH^, Cur-Ag^Tyr^, and Cur-Ag^Qrc^ nanoparticles, and it was confirmed employing zeta (ζ-) potential measurements of the nanoparticles.

As shown in **Table 1**, the zeta (ζ) -potential and DLS measurements were applied to assess the surface charge and hydrodynamic radii (R_hyd_) of synthesized Cur-Ag, Cur-Ag^INH^, Cur-Ag^Tyr^, and Cur-Ag^Qrc^ nanoparticles. The effective surface charge and hydrodynamic radius of nanoparticles play a central role in determining the aggregation states of nanoparticles. Cur-Ag nanoparticles showed ζ-potential values of -26.5 mV, which further changed after their surface functionalization. For Cur-Ag^INH^, Cur-Ag^Tyr^, and Cur-Ag^Qrc^ nanoparticles, the effective ζ-potential values are found to be -26.7 mV, -28.4 mV, and -25.1 mV, correspondingly (**Table 1**). The reported high negative ζ-potential values of the nanoparticles are responsible for electrostatic repulsion between the nanoparticles, and that is why these nanoparticles are highly stable [34]. The DLS studies confirmed the hydrodynamic size of nanoparticles, which was found to be 23.83 nm, 30.13 nm, 30.32 nm, and 32.71 nm for Cur-Ag, Cur-Ag^INH^, Cur-Ag^Tyr^, and Cur-Ag^Qrc^ nanoparticles, respectively. The hydrodynamic size of Cur-Ag^INH^, Cur-Ag^Tyr^, and Cur-Ag^Qrc^ show a percentage increase of ~26.43%, ~27.23%, and ~37.26% for Cur-Ag^INH^, Cur-Ag^Tyr^, and Cur-Ag^Qrc^ nanoparticles concerning Cur-Ag nanoparticles. The increase in the hydrodynamic size of post-functionalized nanoparticles (Cur-Ag^INH^, Cur-Ag^Tyr^, or Cur-Ag^Qrc^) confirmed the surface modification of Cur-Ag nanoparticles.

**Table 1:** Hydrodynamic size and ζ- potential values of Cur-Ag, Cur-Ag^INH^, Cur-Ag^Tyr,^ and Cur-Ag^Qrc^ nanoparticles.

Sample	R_hyd_ (nm)	ζ-values (mV)
Cur-Ag nanoparticles	23.83	-26.5 ± 0.551
Cur-Ag^INH^ nanoparticles	30.13	-26.7 ± 0.208
Cur-Ag^Tyr^ nanoparticles	30.32	-28.4 ± 1.500
Cur-Ag^Qrc^ nanoparticles	32.71	-25.1 ± 0.321

Further, FTIR was employed to identify the presence of different functional groups ​ on the surface of nanoparticles and to confirm the capping of curcumin, isoniazid, tyrosine, or quercetin molecules on nanoparticles (**Fig 2C**). As shown in **S2 Fig** and **S1 Table in S1 File**, the curcumin shows vibrational frequencies at ~1272 cm^-1^, ~1636 cm^-1^, and ~2114 cm^-1^. Due to the C-O stretching of the phenolic group, C = O stretching of conjugated ketone, and symmetric vibration of the CH_3_ group [44]. Likewise, pristine isoniazid (**S2 Fig in S1 File**) exhibited its unique vibrational frequencies at ~1435 cm^-1^ due to C-N stretching or -NH bending, ~1634 cm^-1^ for C = O stretching, ~1216 cm^-1^ due to C-OH (phenolic OH) stretching, ~1228 cm^-1^ because of N-N single bond, ~1417 cm^-1^ due to pyridine ring, ~1488 cm^-1^ for N-O stretching, and ~1557 cm^-1^ due to N-H bending [45]. In tyrosine, the frequencies are found to be at ~1217 cm^-1^, ~1229 cm^-1^, ~1558 cm^-1^, ~1633 cm^-1^, and ~1737 cm^-1^ originating from the C-OH (phenolic) stretching, N-N single bond, N-H bending, carbonyl stretching vibration from the carboxylate ion, and C = O stretching mode, respectively [46, 47]. Similarly, for quercetin molecule show its fundamental vibrational frequencies at ~1170 cm^-1^, ~1229 cm^-1^ or ~1267 cm^-1^, and ~1525 cm^-1^ assigned to stretching of the benzene ring, C-O stretch of phenolic OH (-C-O-H) group and C = C stretching. All the nanoparticles (Cur-Ag, Cur-Ag^INH^, Cur-Ag^Tyr^, and Cur-Ag^Qrc^) reveal distinctive vibrational frequencies with some changes for curcumin, isoniazid, tyrosine, or quercetin molecules on individual nanoparticles, which confirms the presence of the corresponding molecule on the surface of Ag nanoparticles. All the critical vibrational frequencies and the groups they originate from are shown in **S1 Table in S1 File** for all the nanoparticles.

After confirming the physicochemical characteristics of Cur-Ag, Cur-Ag^INH^, Cur-Ag^Tyr^, and Cur-Ag^Qrc^ nanoparticles, they were subjected to investigate their potential biological properties, including %RSC, haemocompatibility, and cell viability/anti-inflammatory effects using mouse Raw 264.7 macrophages.

**Fig 3:**
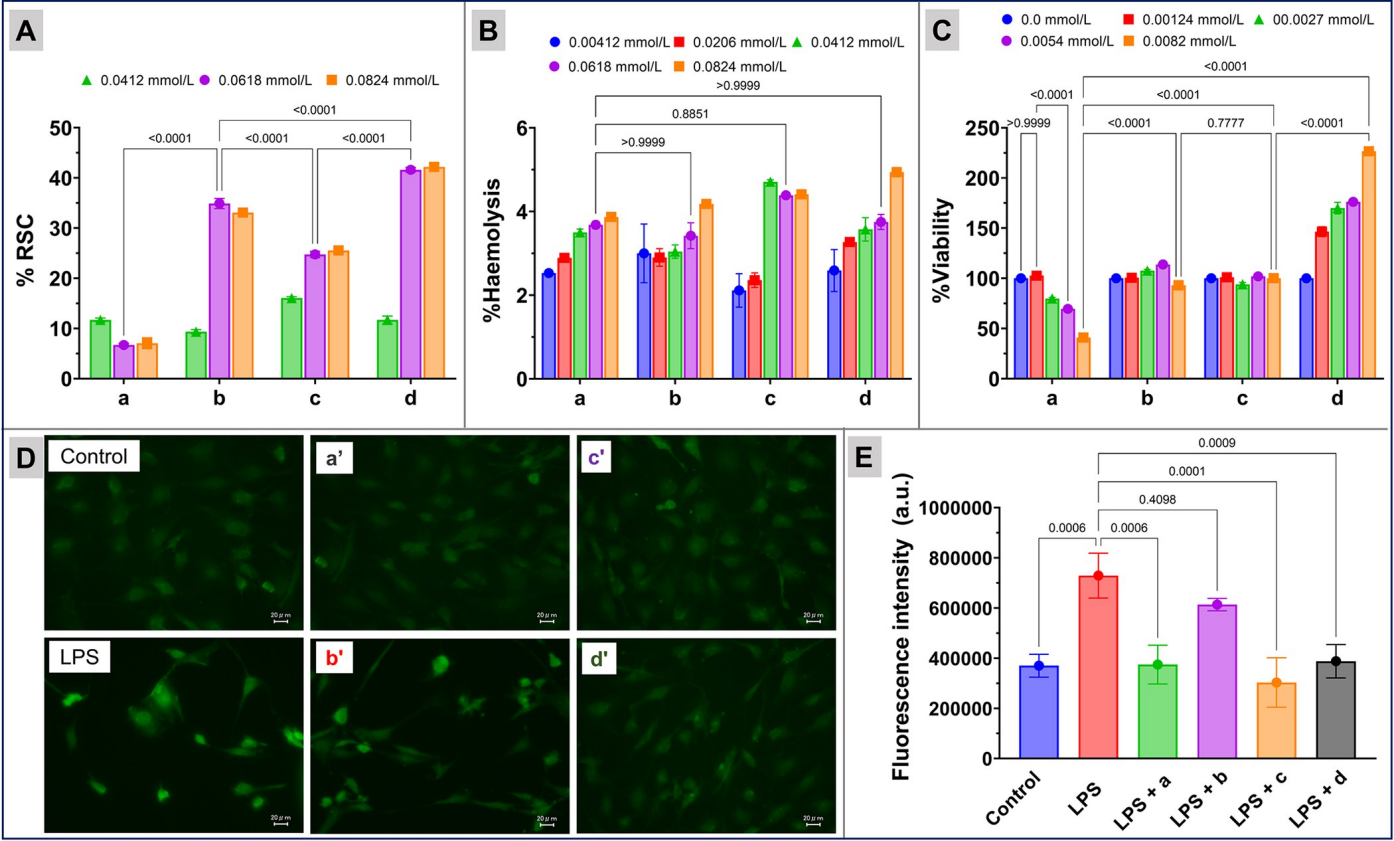
Radical scavenging capacity (RSC) and toxicity of nanoparticles: Panel A: %RSC. Panel B: Haemocompatibility (% Haemolysis). Panel C: %cell viability of mouse RAW 264.7 macrophages treated with Cur-Ag (a), Cur-Ag^INH^ (b), Cur-Ag^Tyr^ (c), and Cur-Ag^Qrc^ (d) nanoparticles. Panel D: generation of ROS by 2’,7’-dichlorodihydrofluorescein diacetate assay in mouse macrophage cells co-treated with lipopolysaccharides (LPS) and nanoparticles. Panel E: relative fluorescent intensity. In Panels D and E, the a’, b’, c’, and d’ represent co-treatment of LPS with Cur-Ag, Cur-Ag^INH^, Cur-Ag^Tyr^, or Cur-Ag^Qrc^ nanoparticles, respectively. In Panels A, B, and C, the results are expressed as mean ± SE, whereas in Panel E, the results are expressed as mean ± SD. The data shown is a compilation of three independent trials. P-values are shown above the square brackets; p < 0.05 was considered statistically significant.

The ABTS assay is one of the most widely used synthetic radical assays for estimating antioxidant activity. ABTS is first oxidized by potassium persulphate to generate cation radicals of ABTS^.+^, which are formed by emitting one electron from the nitrogen atom. The produced blue-green color of radicals can be measured spectroscopically at 734 nm. The ABTS^.+^ reacts with antioxidant molecules leading to the decolorization of the solution, and the calculated % RSC is directly related to the antioxidant activity of the compounds [48, 49]. RSC of Cur-Ag, Cur-Ag^INH^, Cur-Ag^Tyr^, and Cur-Ag^Qrc^ nanoparticles was tested at three different doses 0.0412 mmol/L, 0.0618 mmol/L, and 0.0824 mmol/L, as shown in **Fig 3A**. Cur-Ag nanoparticles show antioxidant behavior like the study reported earlier. The antioxidant nature is derived from the hydrogen in the phenolic or central methylene groups [50, 51]. Post-functionalization leads to an increase in activity for Cur-Ag^INH^, Cur-Ag^Tyr^, or Cur-Ag^Qrc^ nanoparticles. The order of increasing activity was Cur-Ag^Qrc^> Cur-Ag^INH^> Cur-Ag^Tyr^> Cur-Ag nanoparticles. The maximum activity of Cur-Ag^Qrc^ nanoparticles is due to their excellent antioxidant nature, which strongly influences scavenging of free radicals by donating hydrogen atoms or an electron to the free radicals [52]. The antioxidant activity of the flavonoids depends on their functional group arrangement [53] on the surface of nanoparticles. A comparison of 0.0618 mmol/L Ag concentration of all the nanoparticles with the statistical analysis is also shown in Fig 3A, which indicates the significant %RSC of Cur-Ag^Qrc^ nanoparticles (p<0.0001). In similar experimental conditions, curcumin, isoniazid, tyrosine, quercetin, and AgNO_3_ were also tested for their RSC, as displayed in **S3 Fig in S1 File**. The isoniazid showed negative values indicating a negative impact on the RSC due to inducing free radicals, which then exert toxicity to the cells. However, free AgNO_3_, curcumin, tyrosine, and quercetin have shown RSC activity because of their inherent nature, and the activity of these molecules was found to be lesser than those of synthesized nanoparticles [54–56].

Nanoparticle exposure can lead to cellular injuries, nucleic acid damage, cardiovascular diseases, and anemia [57, 58]. In the body, blood is the most common circulating fluid; hence, nanoparticle exposure is inevitable [59]. Thus, it is essential to evaluate the probable toxic effects of synthesized nanoparticles on RBCs. In this context, nanoparticles’ different sizes and dose-dependent activity have been evaluated for toxicity toward blood [57]. Further, it has been established that using different capping and stabilizing agents can reduce nanoparticles’ toxicity [60]. Furthermore, the rupturing of RBCs after nanoparticle treatment can release its internal component into the surrounding fluid that can be visually detected by showing a red color [61]. Therefore, a haemocompatibility assay was performed to evaluate the toxic effects of Cur-Ag, Cur-Ag^INH^, Cur-Ag^Tyr^, and Cur-Ag^Qrc^ nanoparticles on RBCsby selecting four different concentrations: 0.00412 mmol/L, 0.0206 mmol/L, 0.0412 mmol/L, 0.0618 mmol/L, and 0.0824 mmol/L (**Fig 3B)**. The %haemolysis increased with the concentration of nanoparticles in a dose-dependent manner (p < 0.05). Whereas a comparison of Cur-Ag, Cur-Ag^INH^, Cur-Ag^Tyr^, and Cur-Ag^Qrc^ nanoparticles at 0.0618 mmol/L Ag concentration shows significantly higher haemolysis for Cur-Ag^INH^ and Cur-Ag^Qrc^ with respect to pristine Cur-Ag nanoparticles (p>0.9999). However, it is important to state that less than 5% haemolysis was observed at all the evaluated concentrations, confirming the haemocompatibility of the Cur-Ag, Cur-Ag^INH^, Cur-Ag^Tyr^, and Cur-Ag^Qrc^ nanoparticles. Similarly, the effects of pristine AgNO_3_, curcumin, isoniazid, tyrosine, and quercetin were also assessed on RBCs (**S4 Fig in S1 File**).

Further, cell viability studies were conducted on mouse Raw 264.7 macrophages to evaluate the toxic effects of nanoparticles on cells. The MTT assay was performed to assess the cell viability dose-dependent for all the synthesized nanoparticles. The MTT assay is based on the conversion ability of yellow tetrazolium MTT salt into blue MTT formazan crystals by the mitochondrial succinate dehydrogenase enzyme of living cells. Viable cells take up the dye and pass it into mitochondria, which are then solubilized to release MTT formazan, and it can be measured spectrophotometrically at 570 nm to confirm viability [62, 63]. As shown in **Fig 3C**, the assay performed on mouse Raw 264.7 macrophages using different concentrations 0.00 mmol/L, 0.0011 mmol/L, 0.0027 mmol/L, 0.0054 mmol/L, and 0.0082 mmol/L of respective nanoparticles. For the concentration of 0.00 mmol/L, the viability is considered to be 100%. The viability seems to be varied with the surface modification. Cur-Ag nanoparticles at 0.0011 mmol/L concentration showed ~100% viability, which drops significantly to ~79%, ~69%, and ~40% with the increasing doses. Cur-Ag^INH^ and Cur-Ag^Tyr^ nanoparticles demonstrated good cell viability at all the evaluated doses. The Cur-Ag^Qrc^ nanoparticles indicated the highest viability (p<0.0001), and at 0 the upper evaluated dose 0.0082 mmol/L, it shows over ~200% cell viability, which may be attributed to the quercetin surface corona. The results demonstrate the nanoparticles’ biocompatibility and the importance of their surface functionalization.

Nevertheless, it has been reported that Ag nanoparticles can induce toxic effects on cells by inhibiting cell growth, cell morphology changes, and ROS production [64]. These ROS underlie oxidative stress, which damages the intracellular structures, including mitochondria, and DNA ribosomes, ultimately leading to cell death [65, 66]. Therefore, to further assess the effect of nanoparticles’ potential toxicity on different organelles via ROS production, *in vitro* DCFH-DA assay was performed [67]. DCFH-DA is taken up by the cells, and the presence of the cellular esterase cleaves the acetyl groups, leading to the form of DCFH. Further oxidation of DCFH by ROS converts the molecule to DCF, which emits green fluorescence at an excitation wavelength of 485 nm, and an emission wavelength of 530 nm [68]. The effects of Cur-Ag, Cur-Ag^INH^, Cur-Ag^Tyr^, and Cur-Ag^Qrc^ nanoparticles of 0.0054 mmol/L concentration were assessed on mouse Raw 264.7 macrophages, as shown in **Fig 3D**. The treatment of Cur-Ag, Cur-Ag^Qrc^, and Cur-Ag^Tyr^ nanoparticles showed mild emission of green fluorescence when co-treated with lipopolysaccharides (LPS).

In contrast, Cur-Ag^INH^ nanoparticles show some fluorescence, indicating that the formulated Cur-Ag, Cur-Ag^Qrc,^ and Cur-Ag^Tyr^ nanoparticles are nontoxic to the internal organelles and provide a protective effect to LPS-treated macrophages (**Fig 3D**). However, LPS-Cur-Ag^INH^ nanoparticles showed slight fluorescence compared to all other LPS co-treated nanoparticles, indicating their lesser protective impact, which can be attributed to the antibiotic surface corona of nanoparticles. The relative fluorescence intensity of these nanoparticles co-treated with LPS is also evaluated (**Fig 3E**), and from the results, it can be concluded that the low impact of nanoparticles on the cells can be attributed to their specific surface biomolecules.

**Fig 4:**
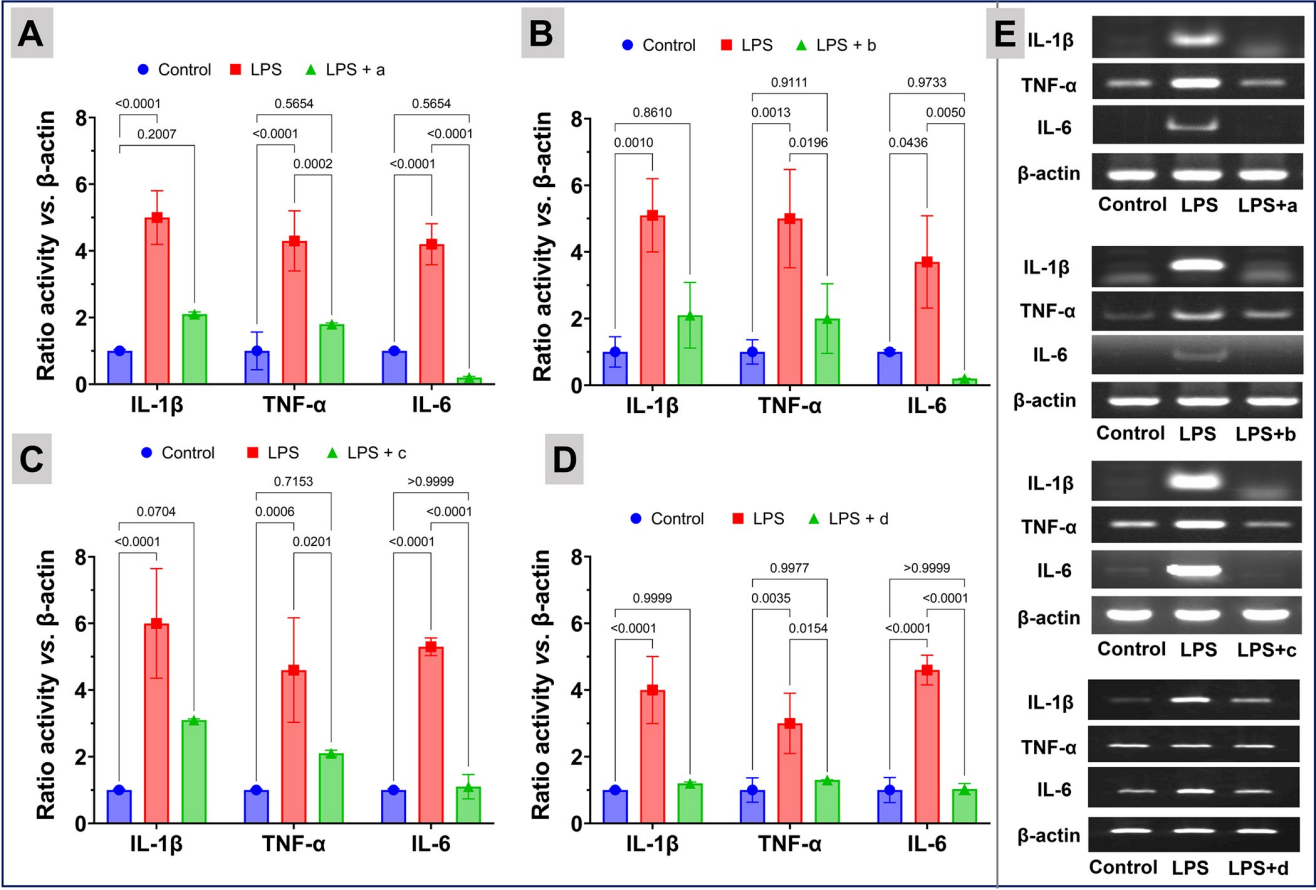
Impact of the nanoparticles on transcription of pro-inflammatory genes: Co-treatment of Cur-Ag (Panel A), Cur-Ag^INH^ (Panel B), Cur-Ag^Tyr^ (Panel C), and Cur-Ag^Qrc^ (Panel D) nanoparticles with lipopolysaccharides (LPS) on the activation of pro-inflammatory cytokines (TNF-α, IL-6, and IL-1β) expression on mouse Raw 264.7 macrophages; β-actin acts as a standard. In Panels A, B, C, and D, the results are expressed as mean ± SD. The data shown is a compilation of three independent trials. P-values are shown above the square brackets; p < 0.05 was considered statistically significant.

**Fig 5:**
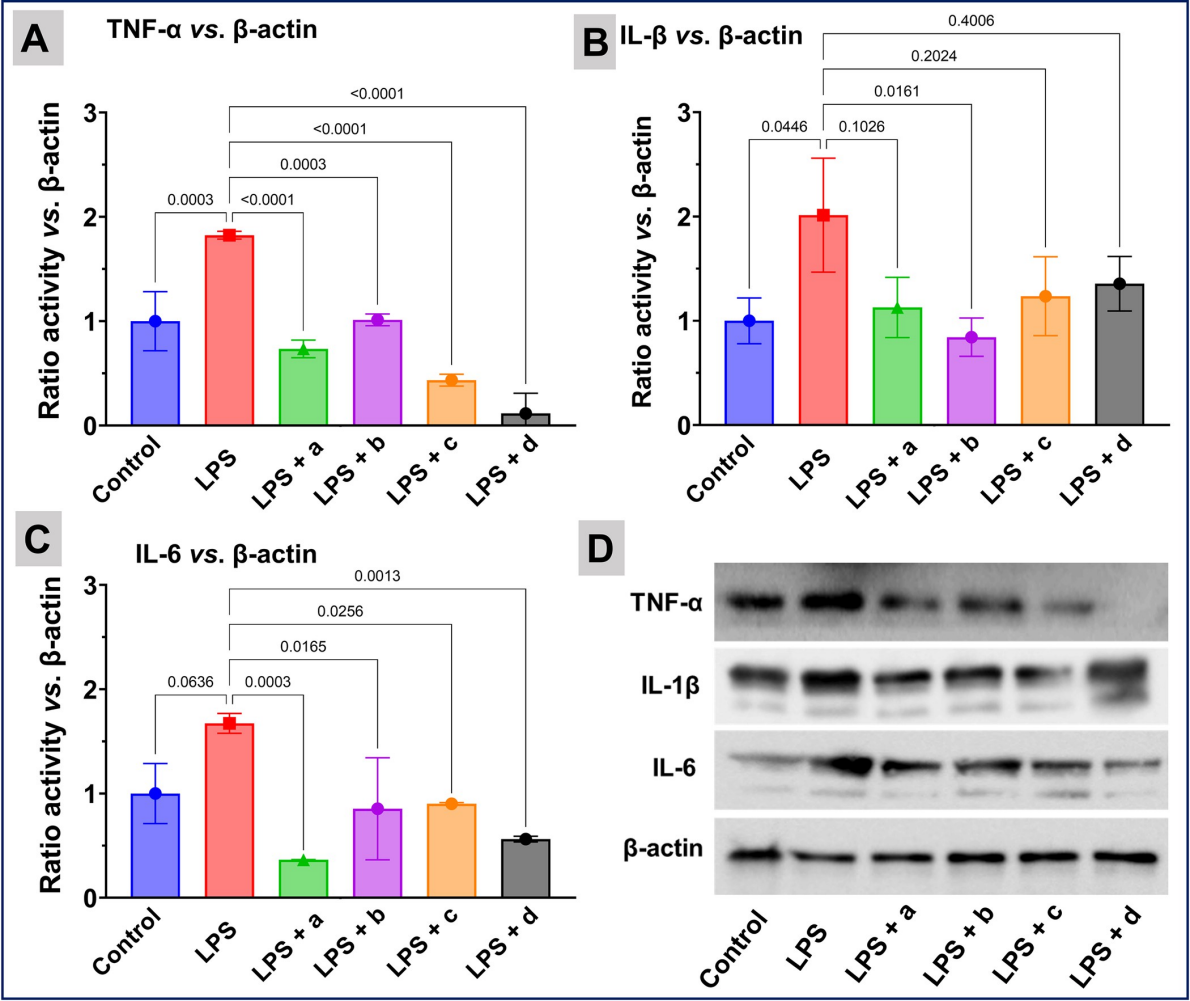
Impact of the nanoparticles on protein expression of TNF-α, IL-6, and IL-1β. The experiments were carried out with mouse Raw 264.7 macrophages by Western blotting after co-treatment with LPS and Cur-Ag (Panel A), Cur-Ag^INH^ (Panel B), Cur-Ag^Tyr^ (Panel C), or Cur-Ag^Qrc^ (Panel D) nanoparticles, respectively. β-actin acts as a standard. In Panels A, B, and C, the results are expressed as mean ± SD. The data shown is a compilation of three independent trials. P-values are shown above the square brackets; p < 0.05 was considered statistically significant.

Besides cell death, the nanoparticles have been shown to enhance the expression of pro-inflammatory cytokines and activate inflammatory cells, which further increase the generation of ROS in cells [69]. Therefore, the production of pro-inflammatory IL-6, IL-1β, and TNF-α cytokines by co-treatment of 0.0054 mmol/L of Cur-Ag, Cur-Ag^INH^, Cur-Ag^Tyr^, and Cur-Ag^Qrc^ nanoparticles with LPS is assessed. LPS is well known to stimulate the production of pro-inflammatory IL-6, IL-1β, and TNF-α. As illustrated in **Fig 4**, the treatment of mouse 264.7 macrophages with LPS increased the transcription of pro-inflammatory genes. However, the co-treatment of macrophages with Cur-Ag, Cur-Ag^INH^, Cur-Ag^Tyr^, or Cur-Ag^Qrc^ nanoparticles caused a significant decrease in the transcription of pro-inflammatory cytokines especially for IL-6. Moreover, the Western blotting was performed for the similar concentrations of Cur-Ag, Cur-Ag^INH^, Cur-Ag^Tyr^, and Cur-Ag^Qrc^ nanoparticles as shown in **Fig 5**. Herein, it is essential to state that the β-actin was used as a housekeeping gene to assess macrophage inflammatory response **(Figs 4 and 5)**.

The expression of pro-inflammatory cytokines protein expression increased with the treatment of LPS. However, co-treatment with the nanoparticles significantly reduced (p<0.0001) the expression of pro-inflammatory cytokine TNF-αas shown in (**Fig 5A)**. This can be attributed to the presence of curcumin, isoniazid, tyrosine, or quercetin biomolecules helps in reduction of pro-inflammatory cytokines (IL-6, IL-1β, and TNF-α). To further validate the observations, pure curcumin, isoniazid, tyrosine, and quercetin molecules were employed in similar experimental conditions, as shown in **S5 and S6 Figs in S1 File**. From **Figs 4** and **5**, it can be clearly concluded that the different surface coronas of the nanoparticles can influence different genes and accordingly up or down-regulate the cytokines expression. In addition, the Ag nanoparticles are reported to have anti-bacterial, anti-fungal, and immunomodulatory activities [70–72].

In contrast, curcumin shows anti-inflammatory action due to the presence of keto form and a double bond in the structure leading to controlling various transcription factors, cytokines, and protein kinases [73]. Moreover, curcumin inhibits the synthesis of inflammatory molecules like TNF-α, which is responsible for producing inflammatory products. It modulates the inflammatory response by down-regulating the expression of cyclooxygenase-2 (COX-2), lipoxygenase (LOX), and phospholipase A2 (PLA2s) enzyme pathway that hinders the inflammatory response mediators [74, 75]. Likewise, free quercetin inhibits the production of TNF-α and nitric oxide (NO) in murine macrophages [76]. The findings confirm that the double functionalized nanoparticles can down-regulate pro-inflammatory genes. By choosing specific biomolecules and nanoparticles, the biomedical potential of the particles can be fine-tuned, and new prospects in nanomedicine can be achieved.

## 4. Conclusion

Hitherto, silver nanoparticles have been used for their anti-bacterial potential, whereas curcumin is a naturally occurring traditional therapeutic agent. However, due to the water-insoluble nature, the full therapeutic benefits of curcumin have not been realized. Recent developments in nanotechnology and the essential role of surface corona wherein organic-inorganic moieties can be conjugated can open new avenues for biology and medicine. Therefore, in the present study, Cur-Ag nanoparticles are synthesized, and a suitable surface corona of isoniazid, tyrosine, and quercetin is engineered around them to develop Cur-Ag^INH^, Cur-Ag^Tyr^, and Cur-Ag^Qrc^ nanoparticles. The impact of the dual surface corona containing curcumin and a functional biomolecule has been evaluated by different *in vitro* biological assays. The interaction of nanoparticles with RBCs, mouse RAW 264.7 macrophages, and pro-inflammatory cytokines confirm their haemocompatibility, cytocompatibility, and anti-inflammatory properties. Among the Cur-Ag, Cur-Ag^INH^, Cur-Ag^Tyr,^ and Cur-Ag^Qrc^ nanoparticles, Cur-Ag^Qrc^ nanoparticles showed relatively higher radical scavenging capacity, positive impact on cell viability and cell proliferation, and lesser generation of reactive oxygen species. Furthermore, the specific surface corona on nanoparticles can influence different genes and accordingly up or down-regulate cytokine expression to control inflammation. The present study indicates the importance of engineered nanoparticle surfaces to unravel their innovative potential for nanomedicine applications. However, additional *in vivo* and experimental model studies must be conducted for further use of such functional nanoparticle systems.

## Supporting information

S1 File (DOCX)

S1 Raw image (DOCX)

## References

[1] Mittal M., Mohammad S. R., Khiem T., Sekhar P. R. and Asrar M. B., Reactive oxygen species in inflammation and tissue injury, Antioxid Redox Signal, 20(7) (2014) 1126–1167, doi: 10.1089/ars.2012.5149 . 23991888 PMC3929010

[2] Maureen D. R., Diana A. and Bates A., Activation of apoptosis signaling pathways by reactive oxygen species, Biochim Biophys Acta Bioenerg, 1863(12) (2016) 2977–2992, doi: 10.1016/j.bbamcr.2016.09.012 . 27646922

[3] He L., He T., Shabnam F., Ji. Linbao, L. Tianyi and M. Xi, Antioxidants maintain cellular redox homeostasis by elimination of reactive oxygen species, Cell. Physiol. Biochem, 44(2) (2017) 532–553, doi: 10.1159/000485089 . 29145191

[4] Duque G. A. and Descoteaux A., Macrophage cytokines: involvement in immunity and infectious diseases, Front. Immunol, 5 (2014) 1–12, doi: 10.3389/fimmu.2014.00491 . 25339958 PMC4188125

[5] Prabhu S., and Poulose E. K., Silver nanoparticles: mechanism of antimicrobial action, synthesis, medical applications, and toxicity effects, Int Nano Lett, 2 (32) (2012) 1–10, 10.1186/2228-5326-2-32.

[6] Keshari A. K., Ragini S., Payal S. and Virendra Y. B., Antioxidant and antibacterial activity of silver nanoparticles synthesized by Cestrum nocturnum, J Ayurveda Integr Med, 11(1) (2020) 37–44, doi: 10.1016/j.jaim.2017.11.003 . 30120058 PMC7125370

[7] Kaphle A., Nagraju N. P. and Daima H. K., Contemporary developments in Nanobiotechnology: Applications, toxicity, sustainability and future perspective, in Nanobiotechnology: Human Health and the Environment, (2018)1–34, 10.1201/9781351031585.

[8] Patel G., Chayan P., Srinivas S. P., Mamta K., Nagraju N.P. and Daima H. K., Methods to evaluate to evaluate the toxicity of engineered nanomaterials for biomedical applications: a review. Environ. Chem. Lett., 19(6) (2021) 4253–4274, 10.1007/s10311-021-01280-1.

[9] Umapathi A., Mamta K. and Daima H. K., Engineered nanomaterials for biomedical applications and their toxicity: a review, Environ. Chem. Lett.,19(6) (2021) 445–468, 10.1007/s10311-021-01307-7.

[10] Madhyastha R., Madhyastha H., Nakajima Y., Omura S. and Maruyama M., Curcumin facilitates fibrinolysis and cellular migration during wound healing by modulating urokinase plasminogen activator expression, Pathophysiol Haemost Thromb, 37 (2010) 59–66, doi: 10.1159/000321375 . 21071923

[11] Pandit R. S., Gaikwad S. C., Agarkar G. A., Gade A. K. and Rai M., Curcumin nanoparticles: physico-chemical fabrication and its in vitro efficacy against human pathogens, 3 Biotech, 5(6) (2015) 991–997, doi: 10.1007/s13205-015-0302-9 . 28324406 PMC4624150

[12] Zubair H., Shafquat A., Ahmad A., Mohammad A. K., Patel K. G., Singh S., et al., Cancer chemoprevention by phytochemicals: Nature’s healing touch, Molecules,22(3) (2017) 1–24, doi: 10.3390/molecules22030395 . 28273819 PMC6155418

[13] Sikora E., Bielak-Zmijewska A., Mosieniak G. and Piwocka K., The promise of slow down ageing may come from curcumin, Curr Pharm Des., 16(7) (2010) 884–892, doi: 10.2174/138161210790883507 . 20388102

[14] Ghalandarlaki N., Alizadeh A. M. and Ashkani-Esfahani S., Nanotechnology-applied curcumin for different diseases therapy, Biomed Res. Int, 2014 (2014) 1–23, doi: 10.1155/2014/394264 . 24995293 PMC4066676

[15] Shome S., Talukdar A. D., Choudhury M. D., Bhattacharya M. K. and Upadhyaya H., Curcumin as potential therapeutic natural product: a nanobiotechnological perspective, J. Pharm Pharmacol, 68(12) (2016) 1481–1500, doi: 10.1111/jphp.12611 . 27747859

[16] Soto-Quintero A., Nakane G., olga G. and Isabel Q. G., Curcumin to promote the synthesis of silver NPs and their self-assembly with a thermoresponsive polymer in core-shell nanohybrids, Sci.Rep, 9(1) (2019)1–14, doi: 10.1038/s41598-019-54752-4 . 31796864 PMC6890765

[17] Matur M., Madhyastha H., Shruthi T. S., Madhyastha R., Srinivas S. P., Nagraju N. P., et al., Engineering bioactive surfaces on nanoparticles and their biological interactions, Sci Rep, 10(1) (2020) 1–14, doi: 10.1038/s41598-020-75465-z . 33184324 PMC7665184

[18] Umapathi A., Nagraju N. P., Madhyastha H., Singh M., Madhyastha R., Maruyama M. et al., Curcumin and isonicotinic acid hydrazide functionalized gold nanoparticles for selective anticancer action, Colloids Surf, A: Physicochem and Eng Asp, 607 (2020) 1–10, 10.1016/j.colsurfa.2020.125484.

[19] Madhyastha H., Halder S., Queen Intan N., Madhyastha R., Mohanapriya A., Sudhakaran R., et al., Surface refined Au Quercetin nanoconjugate stimulates dermal cell migration: possible implication in wound healing, RSC Adv, 10(62) (2020) 37683–37694, doi: 10.1039/d0ra06690g . 35515178 PMC9057138

[20] Madhyastha H., Madhyastha R., Abhishek T., Sakai K., Dev A., Singh S., et al., c-Phycocyanin primed silver nano conjugates: Studies on red blood cell stress resilience mechanism. Colloids Surf. B, 194 (2020) 111211, 10.1016/j.colsurfb.2020.111211. 32615521

[21] Daima H. K., Selvakannan P. R., Shukla R., Bhargava S. and Bansal V., Fine-Tuning the Antimicrobial Profile of Biocompatible Gold Nanoparticles by Sequential Surface Functionalization Using Polyoxometalates and Lysine. PLoS ONE, 8(10) (2013) 1–14, doi: 10.1371/journal.pone.0079676 . 24147146 PMC3798406

[22] Daima H. K., Towards fine-tuning the surface corona of inorganic and organic nanomaterials to control their properties at nano-bio interface, School of Applied Sciences, RMIT (2013),1–236.

[23] Umapathi A., Nagaraju N. P., Madhyastha H., Jain D., Srinivas S. P., Rotello V. M., et al., Highly efficient and selective antimicrobial isonicotinylhydrazide-coated polyoxometalate-functionalized silver nanoparticles, Colloids Surf. B, 184 (2019) 110522, doi: 10.1016/j.colsurfb.2019.110522 . 31586898

[24] Nagaraju N. P., Madhyastha H., Madhyastha R., Yuichi N., Maruyama M., Srinivas S. P., et al., Single step formation of biocompatible bimetallic alloy nanoparticles of gold and silver using isonicotinylhydrazide, Mater. Sci. Eng. C, 96 (2019)286–294, doi: 10.1016/j.msec.2018.11.024 . 30606534

[25] Bhilare N. V., Dhaneshwar S. S. and Mahadik K. R., Amelioration of hepatotoxicity by biocleavable aminothiol chimeras of isoniazid: Design, synthesis, kinetics and pharmacological evaluation, World J Hepatol, 10(7) (2018) 496–508, doi: 10.4254/wjh.v10.i7.496 . 30079136 PMC6068850

[26] Fujisawa J., Kikuchi N. and Hanaya M., Coloration of tyrosine by organic-semiconductor interfacial charge-transfer transitions, Chem. Phys. Lett., 664 (2016) 178–183, 10.1016/j.cplett.2016.10.007.

[27] Monnappa K. S., Nikhath F., Shree G. M., Nath K., Nagaraju N. P. and Daima H. K., Influence of amino acid corona, metallic core and surface functionalisation of nanoparticles on their in-vitro biological behaviour, Int J Nanotechnol 14(9–11) (2017) 816–832, 10.1504/IJNT.2017.086766.

[28] Daima H. K., Selvakannan P. R., Kandjani A. E., Shukla R., Bhargava S. and Bansal V., Synergistic influence of polyoxometalate surface corona towards enhancing the antibacterial performance of tyrosine-capped Ag nanoparticles, Nanoscale 6(2) (2014) 758–765, doi: 10.1039/c3nr03806h . 24165753

[29] Salehi B., Machin L., Monzote L., Sharifi-Rad J., Ezzat S. M., Salem M. A., et al., Therapeutic potential of quercetin: New insights and perspectives for human health, ACS Omega, 5(20) (2020) 11849–11872, doi: 10.1021/acsomega.0c01818 . 32478277 PMC7254783

[30] Sun D., Zhang W., Li. Nuan, Z. Zhiwei, Z. Mou, E. Yang, et al., Silver nanoparticles-quercetin conjugation to siRNA against drug-resistant Bacillus subtilis for effective gene silencing: in vitro and in vivo, Mater Sci Eng C Mater Biol Appl, 63 (2016) 522–534, doi: 10.1016/j.msec.2016.03.024 . 27040247

[31] Sánchez-Moreno C., Methods used to evaluate the free radical scavenging activity in foods and biological systems, J.F.s. and t. international. 8(3) (2002) 121–137, 10.1106/108201302026770.

[32] Ugru M. M., Sheshadria S., Jainb D., Madhyastha H., Madhyastha R., Maruyama M., et al., Insight into the composition and surface corona reliant biological behaviour of quercetin engineered nanoparticles, Colloids Surf, A Physicochem Eng Asp. 548 (2018) 1–9, 10.1016/j.colsurfa.2018.03.055.

[33] Angelini G., Pasc A. and Gasbarri C., Curcumin in silver nanoparticles aqueous solution: Kinetics of keto-enol tautomerism and effects on AgNPs, Colloids Surf, A Physicochem Eng Asp. 603 (2020) 1–5.

[34] Sathishkumar M., Sneha K. and Yun Y., Immobilization of silver nanoparticles synthesized using Curcuma longa tuber powder and extract on cotton cloth for bactericidal activity, Bioresour Technol 101(20) (2010) 7958–7965, doi: 10.1016/j.biortech.2010.05.051 . 20541399

[35] Selvakannan P., Swami A., Srisathiyanarayana D., Shirude P. S., Pasricha R., Mandale A. B., et al., Synthesis of aqueous Au core− Ag shell nanoparticles using tyrosine as a pH-dependent reducing agent and assembling phase-transferred silver nanoparticles at the air− water interface, Langmuir, 20(18) (2004) 7825–7836, doi: 10.1021/la049258j . 15323537

[36] Selvakannan P. R., Ramanathan R., Plowman B. J., Sabri Y. M., Daima H. K., PO’ Mullane A., et al., Probing the effect of charge transfer enhancement in off resonance mode SERS via conjugation of the probe dye between silver nanoparticles and metal substrates, Phys. Chem. Chem. Phys., 15(31) (2013) 12920–12929, doi: 10.1039/c3cp51646f . 23812309

[37] Dubey K., Anand B. G., Badhwar R., Bagler G., Nagaraju N. P., Daima H. K., et al., Tyrosine-and tryptophan-coated gold nanoparticles inhibit amyloid aggregation of insulin, Amino acid, 47(12) (2015) 2551–2560, doi: 10.1007/s00726-015-2046-6 . 26193769

[38] El-Seedi H. R., El-Shabasy R. M., Khalifa S. A. M., Saeed A., Shah A., Shah R., et al., Metal nanoparticles fabricated by green chemistry using natural extracts: biosynthesis, mechanisms, and applications, RSC advances 9(42) (2019) 24539–24559, doi: 10.1039/c9ra02225b . 35527869 PMC9069627

[39] Khan M. J., Shameli K., Sazili A. Q., Selamat J. and Kumari S., Rapid green synthesis and characterization of silver nanoparticles arbitrated by curcumin in an alkaline medium, molecules, 24(4) (2019) 1–12, doi: 10.3390/molecules24040719 . 30781541 PMC6412299

[40] Jaiswal V. D. and Dongre P. M., Biophysical interactions between silver nanoparticle-albumin interface and curcumin, J. Pharm. Anal, 10(2) (2020) 164–177, doi: 10.1016/j.jpha.2020.02.004 . 32373388 PMC7193065

[41] Ghosh M., Kundu S., Pyne A., Sarkar N., Unveiling the behavior of curcumin in biocompatible microemulsion and its differential interaction with gold and silver nanoparticles, J. Phys. Chem. C., 124(6) (2020) 3905–3914, 10.1021/acs.jpcc.9b11553.

[42] Antosiewicz J. M. and Shugar D., UV–Vis spectroscopy of tyrosine side-groups in studies of protein structure. Part 2: selected applications, Biophys. Rev., 8(2) (2016) 163–177, doi: 10.1007/s12551-016-0197-7 . 28510057 PMC4884208

[43] Sisa M., Bonnet S. L., Ferreira D. and Westhuizen J. H. V., Photochemistry of flavonoids, Molecules, 15(8) (2010) 5196–5245, doi: 10.3390/molecules15085196 . 20714295 PMC6257713

[44] Dhakal S., Schmidt W. F., Kim M., Xi Tang Y. Peng and K. Chao, Detection of additives and chemical contaminants in turmeric powder using FT-IR spectroscopy, Foods 8(5) (2019) 1–15, doi: 10.3390/foods8050143 . 31027345 PMC6560428

[45] Cheong B. S. and Cho H. G., Enhanced Raman spectrum of Juglone as Ag surface: Is it a simile to that of lawsone? Bull. Korean. Chem. Soc. 13 (1) (2013) 68–74, 10.5012/bkcs.2013.34.1.68.

[46] Ranaldi S., Belle V., Woudstra M., Rodriguez J., Guigliarelli B., sturgis J., et al., Lid opening and unfolding in human pancreatic lipase at low pH revealed by site-directed spin labeling EPR and FTIR spectroscopy, Biochem. J. 48(3) (2009) 630–638, doi: 10.1021/bi801250s . 19113953

[47] Bhat S. A. and Ahmad S., FTIR, FT-Raman and UV–Vis spectral studies of d-tyrosine molecule, J. Mol. Struct., 1105 (2016) 169–177, 10.1016/j.molstruc.2015.10.040.

[48] Lyu Y., Yu M., Liu Q., Zhang Q., Liu Z., Tian Y., et al., Synthesis of silver nanoparticles using oxidized amylose and combination with curcumin for enhanced antibacterial activity, Carbohydr Polym., 230 (2020) 1–31, doi: 10.1016/j.carbpol.2019.115573 . 31887939

[49] Bedlovičová Z., Strapáč I., Baláž M. and Salayová A., A brief overview on antioxidant activity determination of silver nanoparticles, Molecules, 25(14) (2020) 1–24, doi: 10.3390/molecules25143191 . 32668682 PMC7397195

[50] Alves T. F., Chaud M. V., Grotto D., Jozala A. F., Pandit R., Rai M., et al., Association of silver nanoparticles and curcumin solid dispersion: antimicrobial and antioxidant properties., AAPS PharmSciTech, 19(1) (2018) 225–231, doi: 10.1208/s12249-017-0832-z . 28681332

[51] Mukherjee S., Ray G., Gandhi P. and Kar S. K., Nano Curcumin: Making it useful for Human Therapy, J. Nanomed. Nanotechnol, 11(487) (2020) 1–10.

[52] Parhi B., Bharatiya D. and Swain S. K., Application of quercetin flavonoid based hybrid nanocomposites: A review, Saudi Pharm J., 28(12) (2020) 1719–1732, doi: 10.1016/j.jsps.2020.10.017 . 33424263 PMC7783214

[53] Wang Y., Yin Z., Gao L., Sun D., Hu X., Xue L., et al., Curcumin delays retinal degeneration by regulating microglia activation in the retina of rd1 mice, Cell. Physiol. Biochem., 44(2) (2017) 479–493, doi: 10.1159/000485085 . 29145208

[54] Ramamurthy C. H., Padma M., Samadanam I. D. M., Mareeswaran R., Suyavaran A., Kumar M. S., et al., The extra cellular synthesis of gold and silver nanoparticles and their free radical scavenging and antibacterial properties, Colloids Surf B Biointerfaces, 102 (2013) 808–815, doi: 10.1016/j.colsurfb.2012.09.025 . 23107960

[55] Amorati R., Baschieri A., cowden A. and Valgimigli L. The antioxidant activity of quercetin in water solution, Biomimetics, 2(3) (2017) 1–13, doi: 10.3390/biomimetics2030009 . 31105172 PMC6352608

[56] Gülçin I., Comparison of in vitro antioxidant and antiradical activities of L-tyrosine and L-Dopa, Amino acids, 32(3) (2007) 431–438, doi: 10.1007/s00726-006-0379-x . 16932840

[57] Chen L. Q., Fang Li, Ling J., Ding C. Z., Kang B. and Huang C. Z., Nanotoxicity of silver nanoparticles to red blood cells: size dependent adsorption, uptake, and hemolytic activity, Chem. Res. Toxicol, 28(3) (2015) 501–509, doi: 10.1021/tx500479m . 25602487

[58] Choi J., Reipa V., Hitchins V. M., Goering P. L. and Malinauskas R. A., Physicochemical characterization and in V itro hemolysis evaluation of silver nanoparticles, Toxicol Sci, 123(1) (2011) 133–143, 10.1093/toxsci/kfr149. 21652737

[59] de la Harpe K. M., Pierre P. D. K., Yahya E. C., Thashree M., Lisa C. Du. Toit. and Pillay V., The hemocompatibility of nanoparticles: a review of cell–nanoparticle interactions and hemostasis, Cells, 8(10) (2019) 1–25, doi: 10.3390/cells8101209 . 31591302 PMC6829615

[60] Won T. K., Hyun J. W., Ha K. Young L. J. Hyun, Kang P. H., Sungkyun P., et al., Optimizing hemocompatibility of surfactant-coated silver nanoparticles in human erythrocytes, J Nanosci Nanotechnol,12(8) (2012) 6168–6175, doi: 10.1166/jnn.2012.6433 . 22962723

[61] Adahoun M. A., A-Akhras J. M. H., Jaafar M. S. and Bououdina M., Enhanced anti-cancer and antimicrobial activities of curcumin nanoparticles, A.C.N. Biotechnol, 45(1) (2017) 98–107, doi: 10.3109/21691401.2015.1129628 . 26747522

[62] Kamiloglu S., Sari G., Ozdal T. and Capanoglu E. Guidelines for cell viability assays, Front. food nutr. res., 1(3) (2020) 332–349, 10.1002/fft2.44.

[63] Vajrabhaya L.-o. and Korsuwannawong S., Cytotoxicity evaluation of a Thai herb using tetrazolium (MTT) and sulforhodamine B (SRB) assays., J Anal Sci Technol., 9(1) (2018) 1–6, 10.1186/s40543-018-0146-0.

[64] Kim S. and Ryu D. Y., Silver nanoparticle‐induced oxidative stress, genotoxicity and apoptosis in cultured cells and animal tissues., J Appl Toxicol, 33(2) (2013) 78–89, doi: 10.1002/jat.2792 . 22936301

[65] Zhang L., Lingli W., Youbin S. and Kunhui S., Size-dependent cytotoxicity of silver nanoparticles to Azotobacter vinelandii: Growth inhibition, cell injury, oxidative stress and internalization., PLoS One, 13(12) (2018) 1–18, doi: 10.1371/journal.pone.0209020 . 30566461 PMC6300289

[66] Brandelli A., The interaction of nanostructured antimicrobials with biological systems: Cellular uptake, trafficking and potential toxicity. J.F.S. and H. Wellness, 9(1) (2020) 8–20, 10.1016/j.fshw.2019.12.003.

[67] Roesslein M., Hirsch C., Kaiser J-P., Krug H. F. and Wick P., Comparability of in vitro tests for bioactive nanoparticles: a common assay to detect reactive oxygen species as an example., Int J Mol Sci,14(12) (2013) 24320–24337, doi: 10.3390/ijms141224320 . 24351819 PMC3876113

[68] Kim H., and Xue X., Detection of total reactive oxygen species in adherent cells by 2’, 7’-Dichlorodihydrofluorescein diacetate staining, J Vis Exp, (160) (2020) 1–5, doi: 10.3791/60682 . 32658187 PMC7712457

[69] Tyavambiza C., Elbagory A. M., Madiehe A. M., Meyer M. and Meyer S., The Antimicrobial and Anti-Inflammatory Effects of Silver Nanoparticles Synthesised from Cotyledon orbiculata Aqueous Extract, J. Nanomater., 11(5) (2021) 1343 1–19, doi: 10.3390/nano11051343 . 34065254 PMC8160699

[70] Gomathi M., Rajkumar P. V., Prakasam A. and Ravichandran K., Green synthesis of silver nanoparticles using Datura stramonium leaf extract and assessment of their antibacterial activity., Resource-Efficient Technologies, 3(3) (2017) 280–284, 10.1016/j.reffit.2016.12.005.

[71] Mmola M., Roes-Hill M. L., Durrell K., Bolton J. J., Sibuyi N., Meyer M. E., et al., Enhanced antimicrobial and anticancer activity of silver and gold nanoparticles synthesised using Sargassum incisifolium aqueous extracts., Molecules, 21(12) (2016) 1–22, doi: 10.3390/molecules21121633 . 27918447 PMC6273965

[72] Kumawata M., Madhyastha H. K., Singh M., Jain D., Daima H. K., Functional Silver Nanozymes Regulate Cell Inflammatory Cytokines Expression In Mouse Macrophages, Col. Surf. A, (2022) 129294, 10.1016/j.colsurfa.2022.129294.

[73] Rai M., Pandit R., Gaikwad S., Yadav A. and Gade A., Potential applications of curcumin and curcumin nanoparticles: from traditional therapeutics to modern nanomedicine, Nanotechnol. Rev., 4(2) (2015) 161–172. 10.1515/ntrev-2015-0001.

[74] Jurenka J. S., Anti-inflammatory properties of curcumin, a major constituent of Curcuma longa: a review of preclinical and clinical research, Sci. Rev. Altern. Med., 14(2) (2009) 141–53, . 19594223

[75] Karthikeyan A., Senthil N. and Min T., Nanocurcumin: a promising candidate for therapeutic applications. Front. Pharmacol., 11 (2020) 1–24, doi: 10.3389/fphar.2020.00487 . 32425772 PMC7206872

[76] Manjeet R. and Ghosh B., Quercetin inhibits LPS-induced nitric oxide and tumor necrosis factor-α production in murine macrophages. Int J Immunopharmacol, 21(7) (1999) 435–443, doi: 10.1016/s0192-0561(99)00024-7 . 10454017

