# Supplementary material for: Double functionalized haemocompatible silver nanoparticles control cell inflammatory homeostasis
Source: PLoS One. 2022 Oct 21;17(10):e0276296. doi: 10.1371/journal.pone.0276296 (PMC9586410; doi:10.1371/journal.pone.0276296)
Supplement: S1 File — (DOCX) [file pone.0276296.s001.docx]

***Supporting information***

**Double functionalized haemocompatible silver nanoparticles control cell inflammatory homeostasis**

Mamta Kumawat^1^, Harishkumar Madhyastha^2^, Mandeep Singh^3^, Neerish Revaprasadu^4^, Sangly P. Srinivas^5^, Hemant Kumar Daima^1*^

^1^Amity Center for Nanobiotechnology and Nanomedicine (ACNN), Amity Institute of Biotechnology, Amity University Rajasthan, Jaipur, 303002, Rajasthan, India

^2^Department of Cardiovascular Physiology, Faculty of Medicine, University of Miyazaki, Miyazaki, 8891692, Japan

^3^School of Science, RMIT University, Melbourne 3000, Victoria, Australia

^4^Department of Chemistry, University of Zululand, Private Bag X1001, KwaDlangezwa 3880, South Africa

^5^School of Optometry, Indiana University, Bloomington, IN 47405, USA

^*^Corresponding author: Email: hkdaima@jpr.amity.edu; hkdaima@gmail.com,

Phone: +91 8884774863

**Figure S1.** UV-Visible absorbance spectra of pristine curcumin, isoniazid, tyrosine, and quercetin.

*
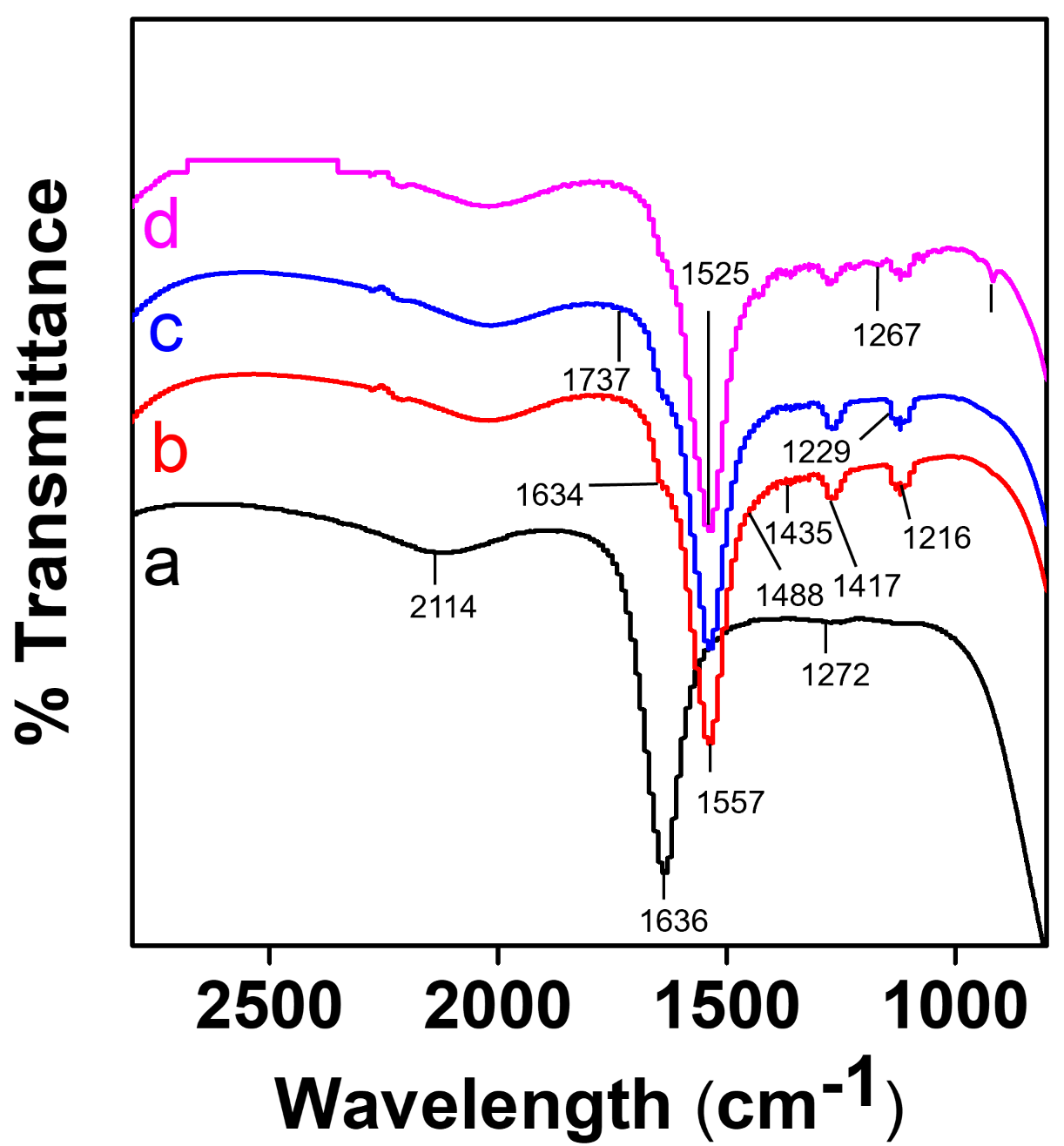
*

**Figure S2.** FTIR vibrational frequencies of pristine curcumin (a), isonicotinic acid hydrazide or isoniazid (b), tyrosine (c), and quercetin (d), respectively.


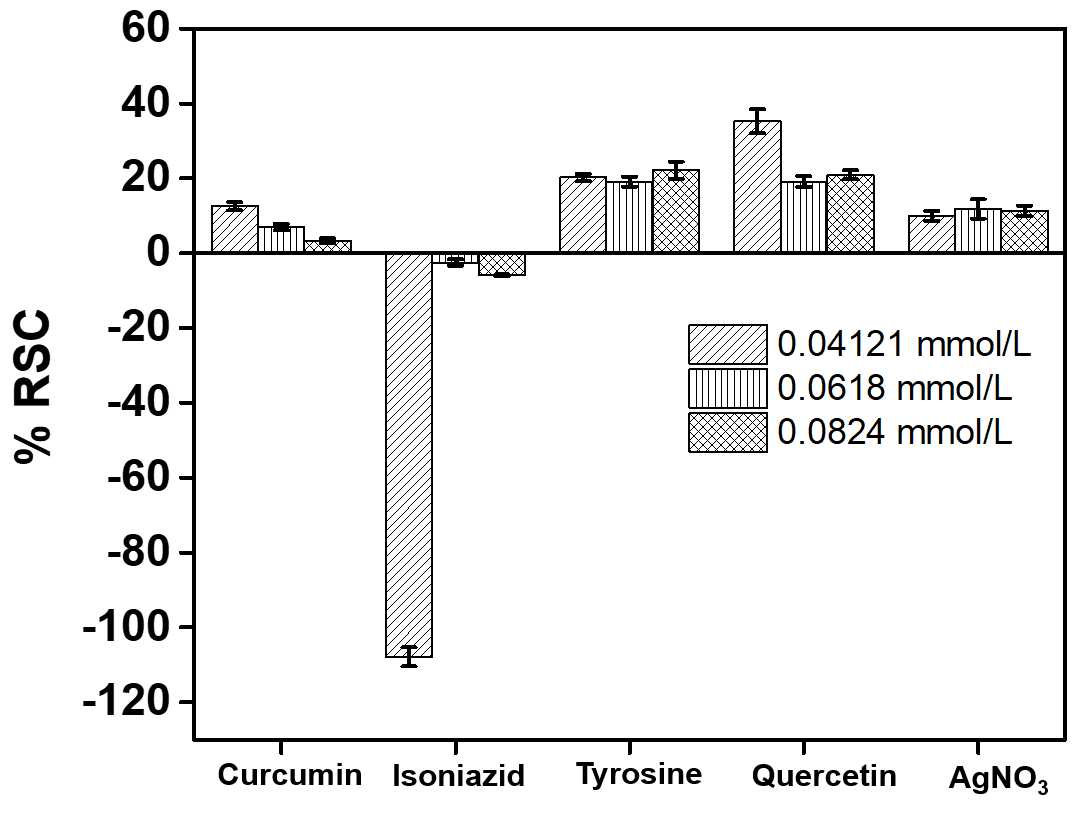


**Figure S3.** The percentage radical scavenging capacity of pristine curcumin, isoniazid, tyrosine, quercetin, and AgNO_3_.


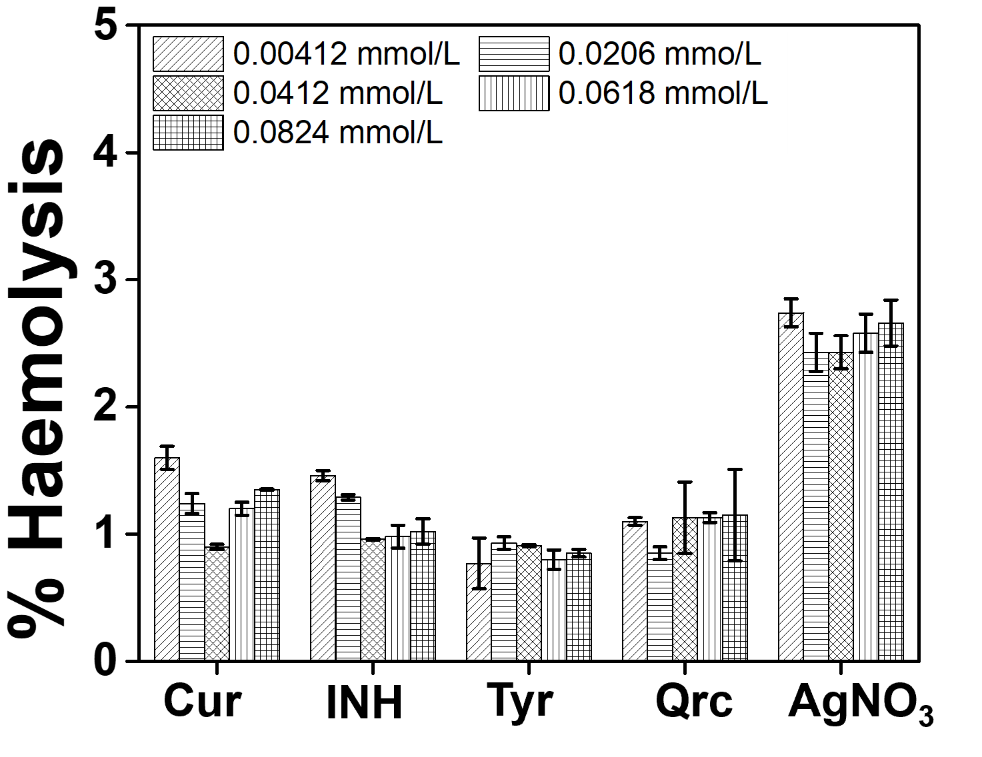


**Figure S4.** Percentage hemolysis activity on RBC’s by varying concentrations of curcumin, isoniazid, tyrosine, quercetin, and AgNO_3_.

**
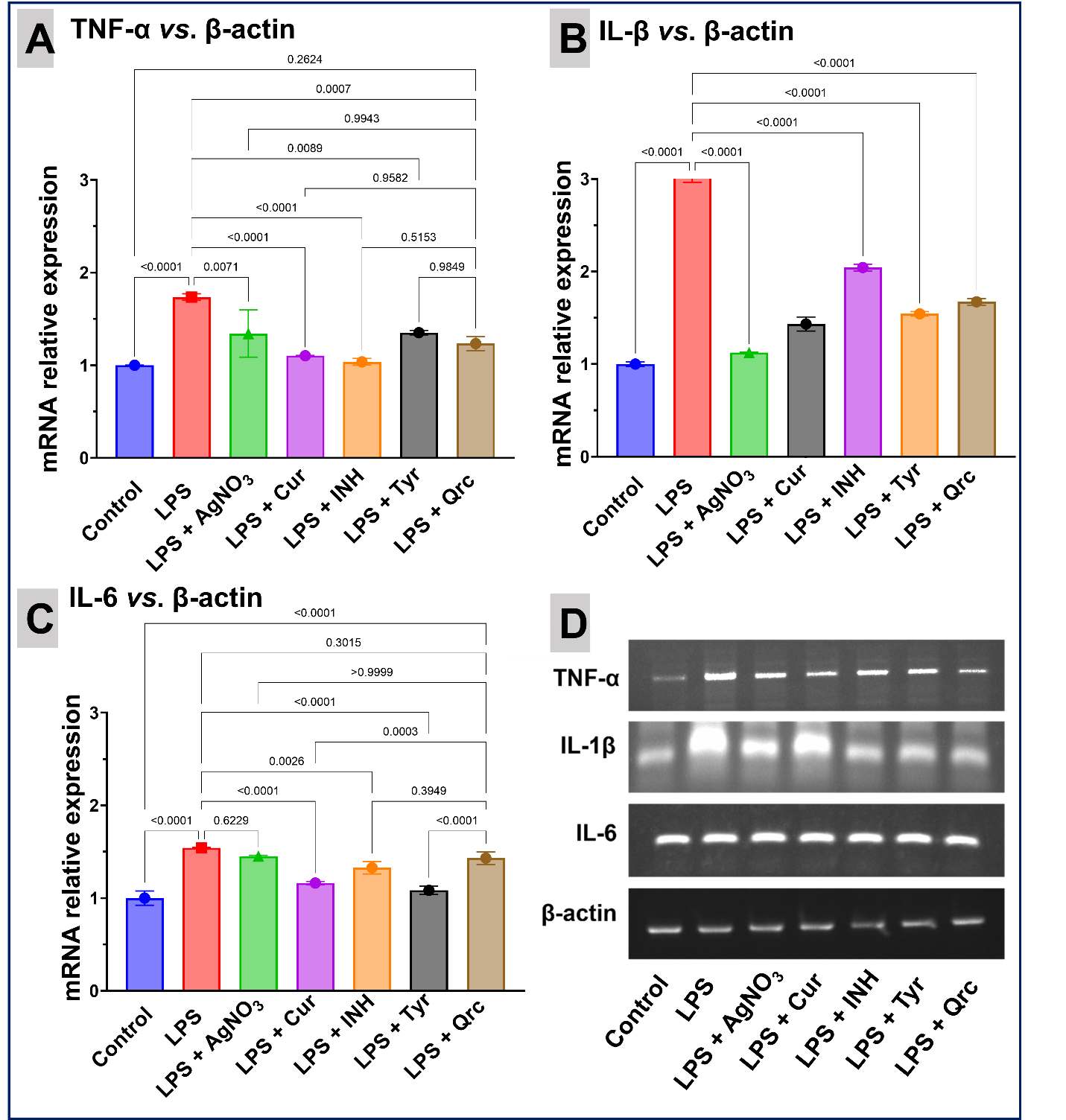
**

**Figure S5.** Co-treatment of curcumin, isoniazid, tyrosine, quercetin, and AgNO_3_ with lipopolysaccharides (LPS) on the activation of pro-inflammatory cytokines (TNF-α, IL-6, and IL-1β) expression on mouse Raw 264.7 macrophages. Herein, β-actin acts as a positive control. The results are expressed as mean ± SD, and the data shown is a compilation of three independent trials. P-values are shown above the square brackets; p < 0.05 was considered statistically significant.

**
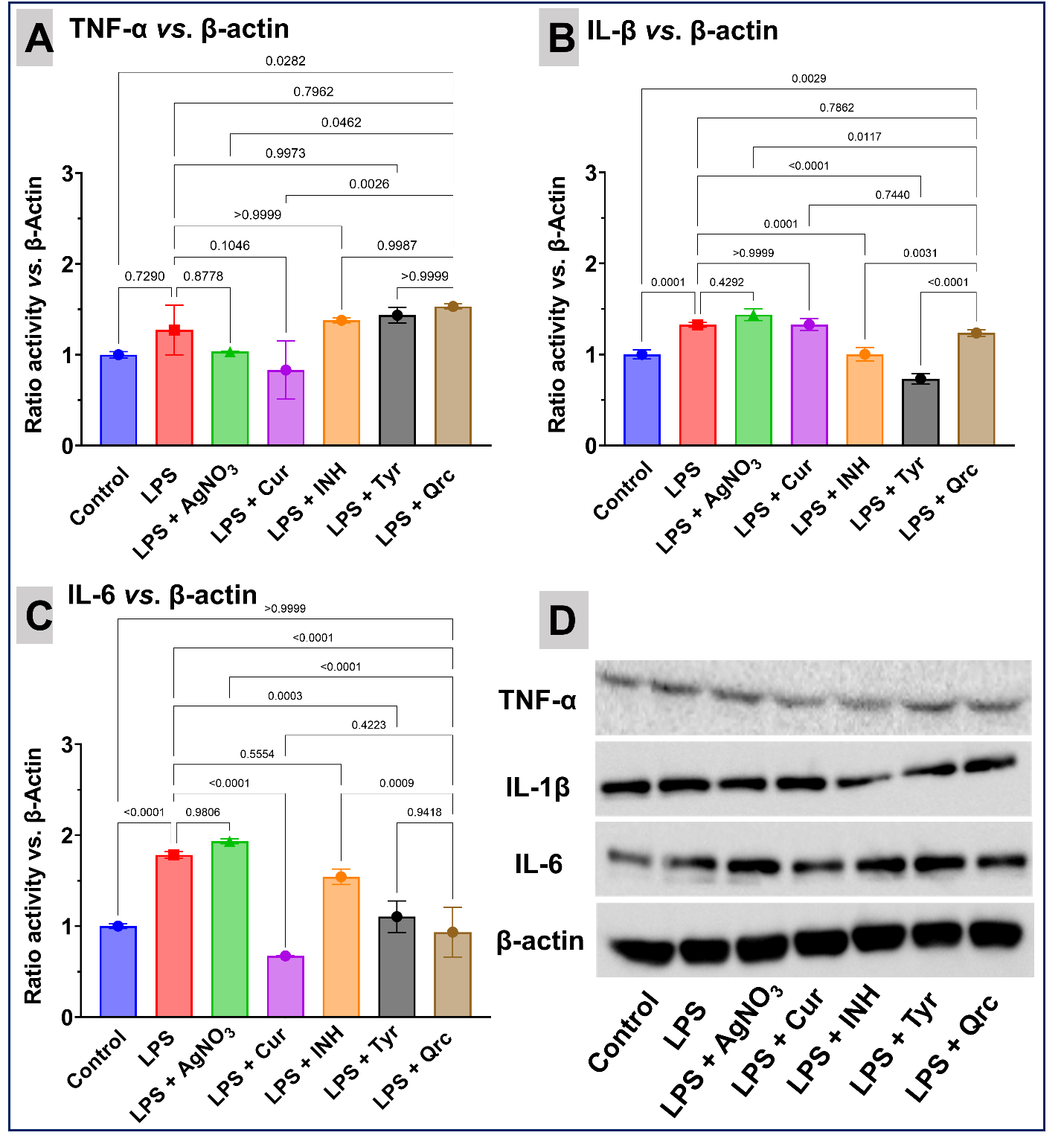
**

**Figure S6.** Assessment of pro-inflammatory cytokines (TNF-α, IL-6, and IL-1β) protein expression in mouse Raw 264.7 macrophages by Western blot assay after co-treatment with lipopolysaccharides (LPS) and curcumin, isoniazid, tyrosine, quercetin, and AgNO_3_. Here, β-actin acts as a positive control. The results are expressed as mean ± SD, and the data shown is a compilation of three independent trials. P-values are shown above the square brackets; p < 0.05 was considered statistically significant.

**Table S1.** The specific vibrational frequencies of pristine curcumin, isoniazid, tyrosine, and quercetin, on the surface of respective Ag nanoparticles.

| **Name** | **Vibrational frequencies** | **Nanoparticle solutions** | **Vibrational frequencies** |
| --- | --- | --- | --- |
| Curcumin | 1271.75 (C-O stretching vibration of the keto-enol form)  1636.08 [carbonyl (C=O) stretching of the conjugated ketone]  2113.77 (symmetric vibration of CH_3_ group) | Cur-Ag  nanoparticles | 1216.74 [C-OH (phenolic OH) stretching]  1228.56 (keto-enol form of curcumin)  1364.80 (O-H bending of phenol)  1455.23 vibration of heptadin chain  1634.75 [carbonyl (C=O) stretching of the conjugated ketone]  2121.84 (symmetric vibration of CH_3_ group) |
| Isoniazid | 1216.73 [C-OH (phenolic OH) stretching]  1228.58 (N-N single bond)  1364.17 (amino group NH_2_ wagging)  1417.59 (pyridine)  1435.20 (C-N stretching/NH bending)  1455.50 [ν(CN)pyridine and eδ(CNH)]  1488.89 (N-O stretching)  1557.89 (N-H bending)  1634.22 (carbonyl group stretching)  1737.57 (C=O stretching)  1867.66 (C=O stretching)  2114.77 (symmetric vibration of CH_3_ group)  2374.88 (O=C=O stretching) | Cur-Ag^INH^ nanoparticles | 1216.73 [C-OH (phenolic OH) stretching]  1228.57 (C-O stretching or keto-enol group)  1364.88 (amino group NH_2_ wagging)  1455.22 (vibration of heptadin chain)  1489.04 (N-O stretching)  1634.56 (carbonyl group C=O stretching)  1737.40 (C=O stretching)  2126.64 (symmetric vibration of CH_3_ group) |
| Tyrosine | 1216.71 [C-OH (phenolic OH) stretching]  1228.50 (N-N single bond)  1364.58 (amino group NH_2_ wagging)  1417.70 (pyridine)  1435.31 (phenolic CO-)  1455.58 [ν(CN)pyridine and eδ(CNH)]  1488.93 (N-O stretching)  1558.08 (N-H bending)  1633.90 (carbonyl stretching vibration from the carboxylate ion)  2121.83 (symmetric vibration of CH_3_ group)  1737.49 (C=O stretching)  1867.61 (C=O stretching)  2114.45 (symmetric vibration of CH_3_ group)  2374.48 (O=C=O stretching) | Cur-Ag^Tyr^  nanoparticles | 1216.74 [C-OH (phenolic OH) stretching]  1228.70 (N-N single bond)  1364.83 (amino group NH_2_ wagging)  1455.60 [ν(CN)pyridine and eδ(CNH)]  1635.20 (formation of quinone structure)  2125.50 (symmetric vibration of CH_3_ group) |
| Quercetin | 1170.15 (stretching of a benzene ring)  1216.70 [C-OH (phenolic OH) stretching]  1228.91{ [C-O stretch of phenolic OH(-C-O-H)]  1266.89}  1318.84 (O-H bending)  1373.53 (O-H bending)  1417.46 (O-H bending)  1435.36 (phenolic CO-)  1455.73 [ν(CN)pyridine and eδ(CNH)]  1525.30 (C=C stretching)  1634.08 (aromatic carbonyl stretching)  1737.55 (C=O stretching)  2114.78 (symmetric vibration of CH_3_ group)  2375.63 (O=C=O stretching) | Cur-Ag^Qrc^  nanoparticles | 1216.72 [C-OH (phenolic OH) stretching]  1228.53 [C-O stretch of phenolic OH(-C-O-H)]  1364.67 (O-H bending)  1417.63 (O-H bending)  1435.10 (phenolic CO-)  1455.32 [ν(CN)pyridine and eδ(CNH)]  1634.44 (aromatic carbonyl stretching)  1737.49 (C=O stretching)  2125.85 (symmetric vibration of CH_3_ group) |
