# Supplementary material for: Double functionalized haemocompatible silver nanoparticles control cell inflammatory homeostasis
Source: PLoS One. 2022 Oct 21;17(10):e0276296. doi: 10.1371/journal.pone.0276296 (PMC9586410; doi:10.1371/journal.pone.0276296)

**TNF**- **α (**mRNA relative expression**)**

Left side panel:

| Lane 1 | Lane 2 | Lane 3 | Lane 4 | Lane 5 | Lane 6 | Lane 7 |
| --- | --- | --- | --- | --- | --- | --- |
| Control | LPS | Cur-Ag | Cur- Ag^INH^ | Cur-Ag^Tyr^ | Cur-Ag^Qrc^ | x |

Right side panel:

| Lane 1 | Lane 2 | Lane 3 | Lane 4 | Lane 5 | Lane 6 | Lane 7 | Lane 8 |
| --- | --- | --- | --- | --- | --- | --- | --- |
| Control | LPS | LPS+  AgNO_3_ | LPS+  curcumin | LPS+  isoniazid | LPS+  Tyrosine | LPS+  Quercetin | x |


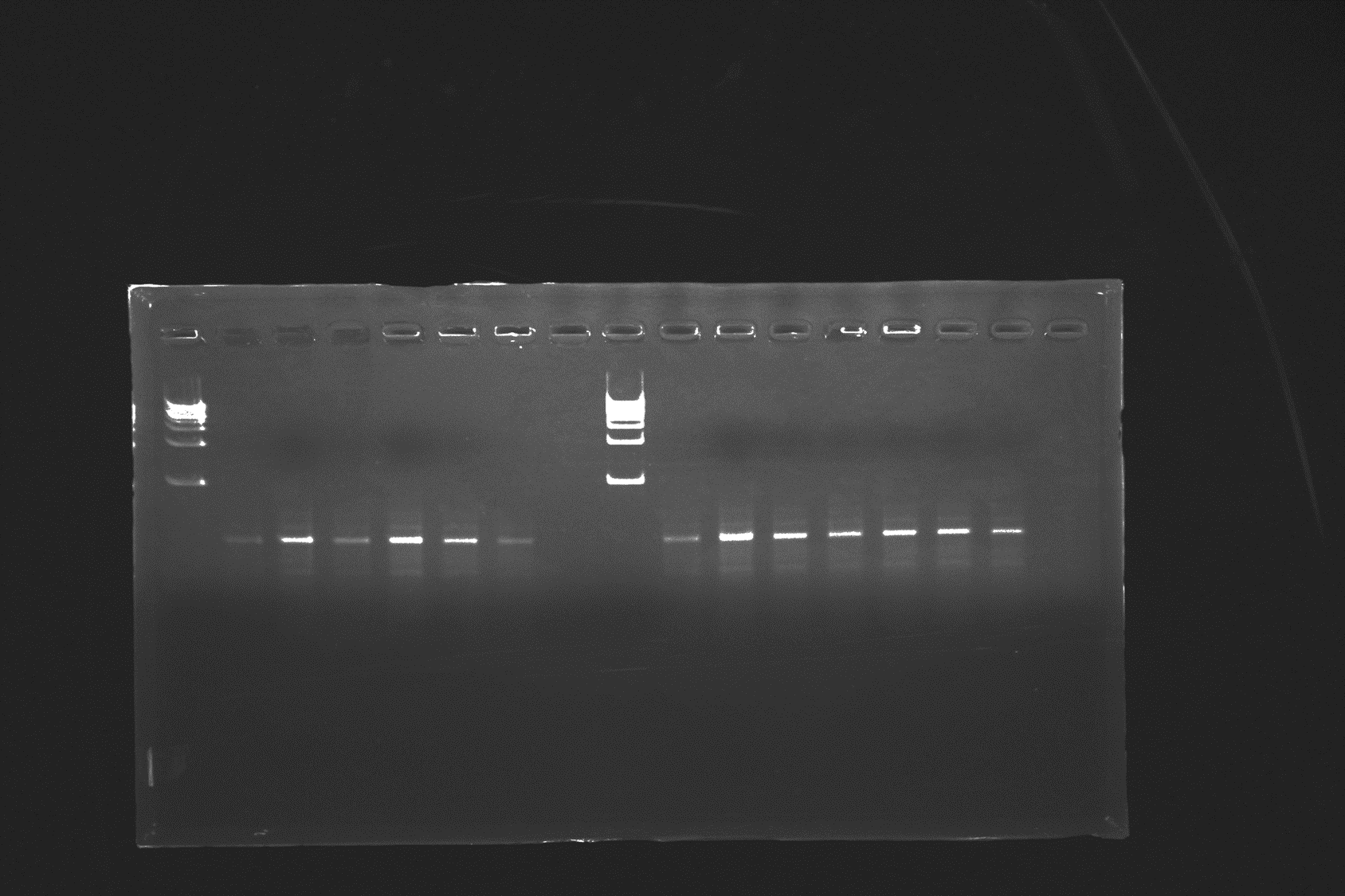


1 2 3 4 5 6 7 1 2 3 4 5 6 7 8

Left side panel

Right side panel

**IL**-**1β**

Left side panel:

| Lane 1 | Lane 2 | Lane 3 | Lane 4 | Lane 5 | Lane 6 | Lane 7 |
| --- | --- | --- | --- | --- | --- | --- |
| Control | LPS | Cur-Ag | Cur- Ag^INH^ | Cur-Ag^Tyr^ | Cur-Ag^Qrc^ | x |

Right side panel:

| Lane 1 | Lane 2 | Lane 3 | Lane 4 | Lane 5 | Lane 6 | Lane 7 | Lane 8 |
| --- | --- | --- | --- | --- | --- | --- | --- |
| Control | LPS | LPS+  AgNO_3_ | LPS+  curcumin | LPS+  isoniazid | LPS+  Tyrosine | LPS+  Quercetin | x |





1 2 3 4 5 6 7 1 2 3 4 5 6 7 8

Right side panel

Left side panel

**IL**-**6**

Left side panel:

| Lane 1 | Lane 2 | Lane 3 | Lane 4 | Lane 5 | Lane 6 | Lane 7 |
| --- | --- | --- | --- | --- | --- | --- |
| Control | LPS | Cur-Ag | Cur- Ag^INH^ | Cur-Ag^Tyr^ | Cur-Ag^Qrc^ | x |

Right side panel:

| Lane 1 | Lane 2 | Lane 3 | Lane 4 | Lane 5 | Lane 6 | Lane 7 | Lane 8 |
| --- | --- | --- | --- | --- | --- | --- | --- |
| Control | LPS | LPS+  AgNO_3_ | LPS+  curcumin | LPS+  isoniazid | LPS+  Tyrosine | LPS+  Quercetin | x |





1 2 3 4 5 6 7 1 2 3 4 5 6 7 8

Right side panel

Left side panel

**β**-**actin**

Left side panel:

| Lane 1 | Lane 2 | Lane 3 | Lane 4 | Lane 5 | Lane 6 | Lane 7 |
| --- | --- | --- | --- | --- | --- | --- |
| Control | LPS | Cur-Ag | Cur- Ag^INH^ | Cur-Ag^Tyr^ | Cur-Ag^Qrc^ | x |

Right side panel:

| Lane 1 | Lane 2 | Lane 3 | Lane 4 | Lane 5 | Lane 6 | Lane 7 | Lane 8 |
| --- | --- | --- | --- | --- | --- | --- | --- |
| Control | LPS | LPS+  AgNO_3_ | LPS+  curcumin | LPS+  isoniazid | LPS+  Tyrosine | LPS+  Quercetin | x |





1 2 3 4 5 6 7 1 2 3 4 5 6 7 8

Right side panel

Left side panel

**TNF**- **α (**Protein relative expression**)**

| **Lane 1** | **Lane 2** | **Lane 3** | **Lane 4** | **Lane 5** | **Lane 6** |
| --- | --- | --- | --- | --- | --- |
| Control | LPS | Cur-Ag | Cur- Ag^INH^ | Cur-Ag^Tyr^ | Cur-Ag^Qrc^ |


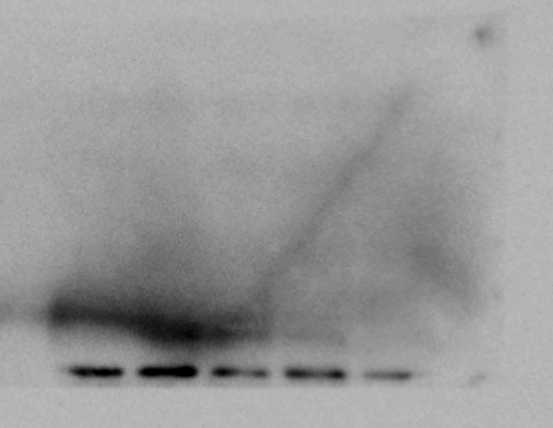


**IL**-**1β**

| **Lane 1** | **Lane 2** | **Lane 3** | **Lane 4** | **Lane 5** | **Lane 6** |
| --- | --- | --- | --- | --- | --- |
| Control | LPS | Cur-Ag | Cur- Ag^INH^ | Cur-Ag^Tyr^ | Cur-Ag^Qrc^ |

**
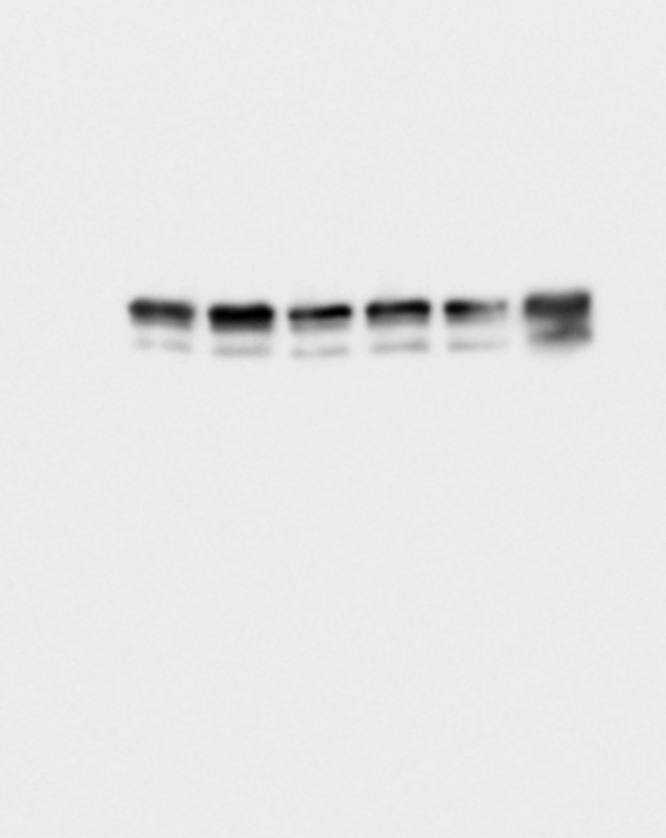
**

**IL**-**6**

| **Lane 1** | **Lane 2** | **Lane 3** | **Lane 4** | **Lane 5** | **Lane 6** |
| --- | --- | --- | --- | --- | --- |
| Control | LPS | Cur-Ag | Cur- Ag^INH^ | Cur-Ag^Tyr^ | Cur-Ag^Qrc^ |


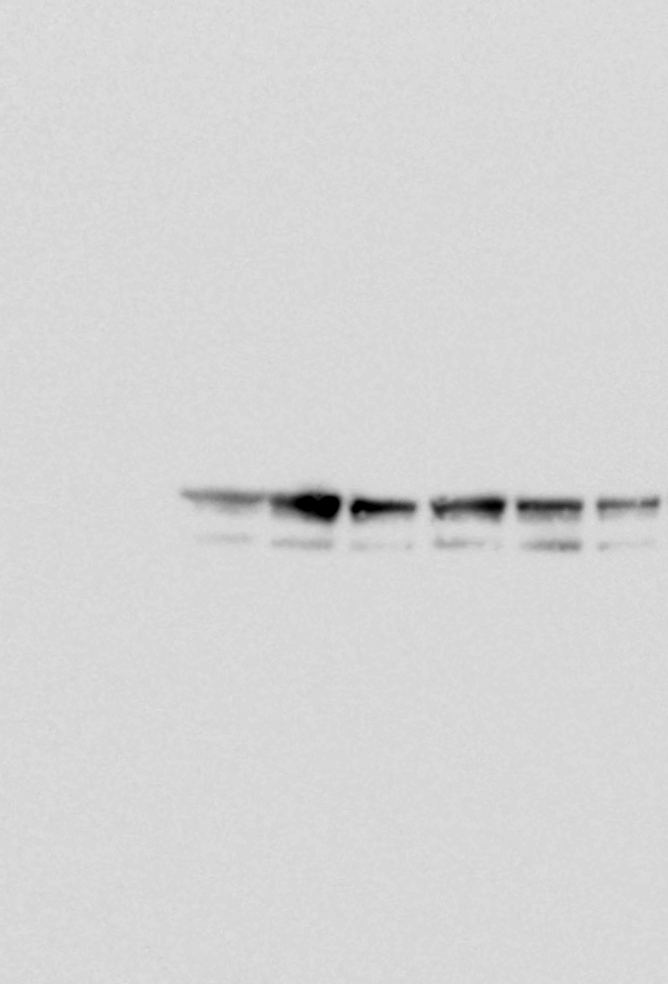


**β**-**actin**

| **Lane 1** | **Lane 2** | **Lane 3** | **Lane 4** | **Lane 5** | **Lane 6** |
| --- | --- | --- | --- | --- | --- |
| Control | LPS | Cur-Ag | Cur- Ag^INH^ | Cur-Ag^Tyr^ | Cur-Ag^Qrc^ |


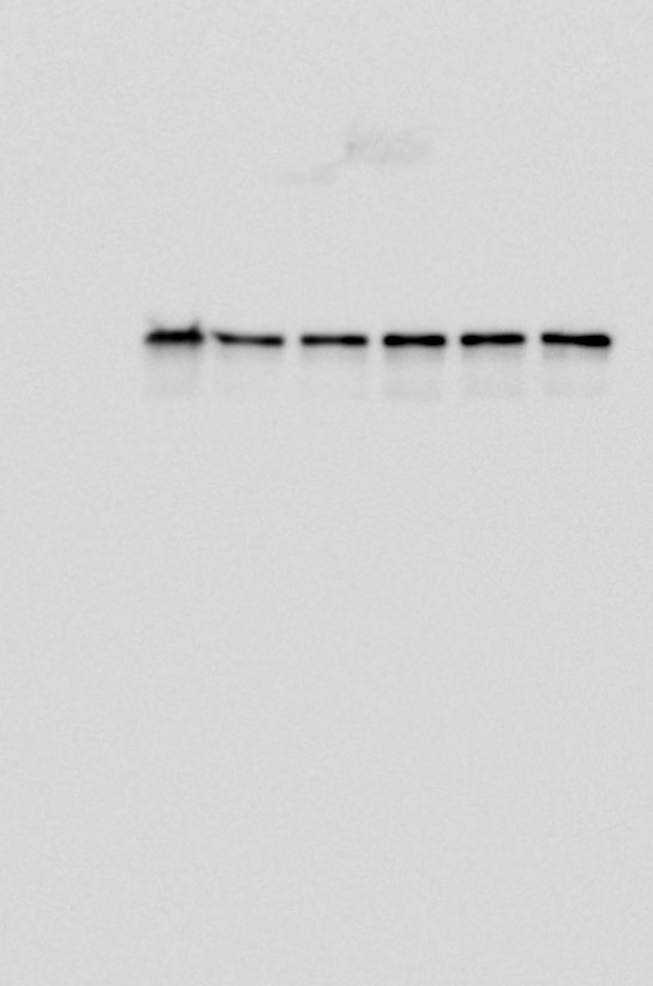


**TNF**- **α (**Protein relative expression**)**

| Lane 1 | Lane 2 | Lane 3 | Lane 4 | Lane 5 | Lane 6 | Lane 7 |
| --- | --- | --- | --- | --- | --- | --- |
| Control | LPS | LPS+  AgNO_3_ | LPS+  curcumin | LPS+  isoniazid | LPS+  Tyrosine | LPS+  Quercetin |





**IL**-**1β**

| Lane 1 | Lane 2 | Lane 3 | Lane 4 | Lane 5 | Lane 6 | Lane 7 |
| --- | --- | --- | --- | --- | --- | --- |
| Control | LPS | LPS+  AgNO_3_ | LPS+  curcumin | LPS+  isoniazid | LPS+  Tyrosine | LPS+  Quercetin |


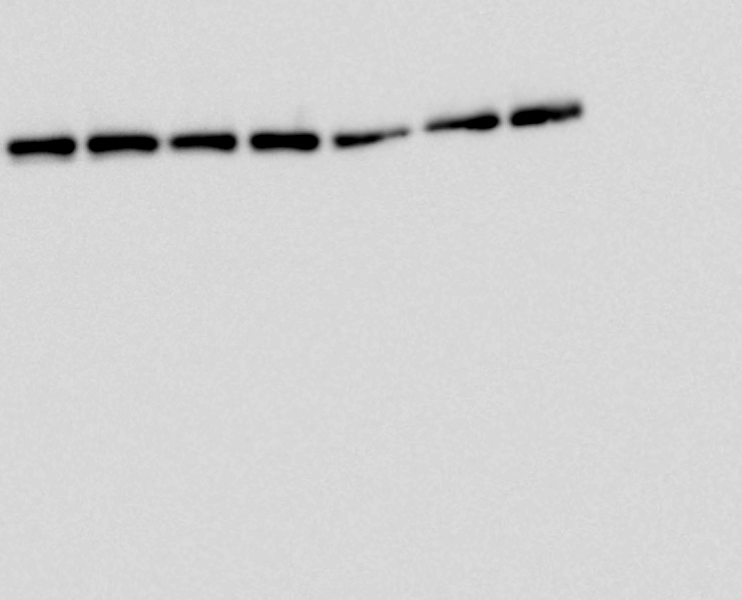


**IL**-**6**

| Lane 1 | Lane 2 | Lane 3 | Lane 4 | Lane 5 | Lane 6 | Lane 7 |
| --- | --- | --- | --- | --- | --- | --- |
| Control | LPS | LPS+  AgNO_3_ | LPS+  curcumin | LPS+  isoniazid | LPS+  Tyrosine | LPS+  Quercetin |


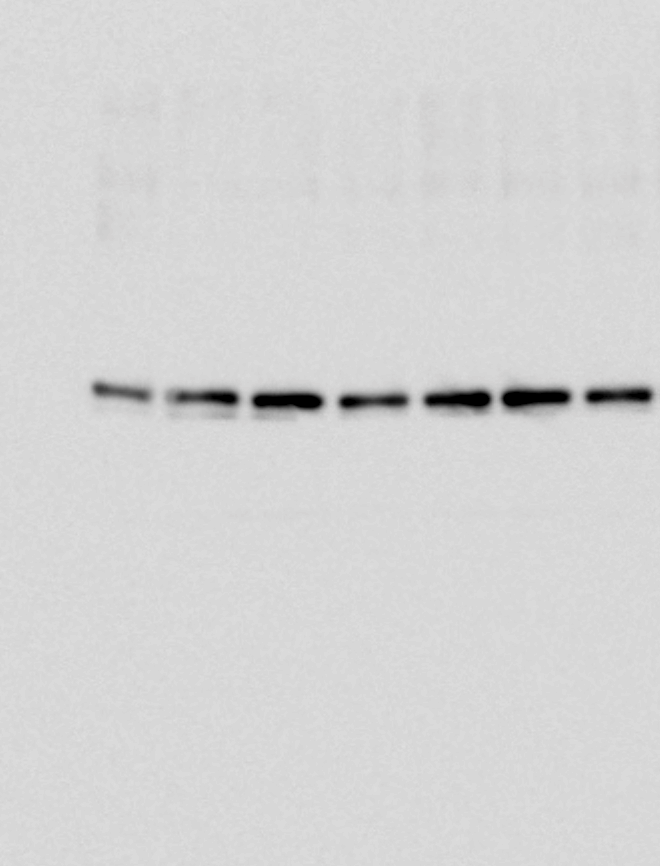


**β**-**actin**

| Lane 1 | Lane 2 | Lane 3 | Lane 4 | Lane 5 | Lane 6 | Lane 7 |
| --- | --- | --- | --- | --- | --- | --- |
| Control | LPS | LPS+  AgNO_3_ | LPS+  curcumin | LPS+  isoniazid | LPS+  Tyrosine | LPS+  Quercetin |


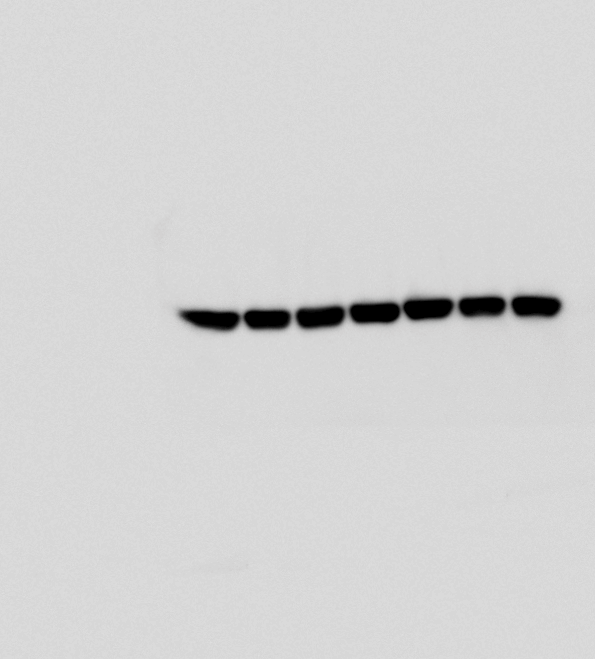

Supplement: S1 Raw image — (DOCX) [file pone.0276296.s002.docx]
